# Joint Analysis of the Epidemic Evolution and Human Mobility During the First Wave of COVID-19 in Spain: Retrospective Study

**DOI:** 10.2196/40514

**Published:** 2023-05-22

**Authors:** Benjamin Steinegger, Clara Granell, Giacomo Rapisardi, Sergio Gómez, Joan Matamalas, David Soriano-Paños, Jesús Gómez-Gardeñes, Alex Arenas

**Affiliations:** 1 Universitat Rovira i Virgili Tarragona Spain; 2 Harvard Medical School Boston, MA United States; 3 Brigham and Women’s Hospital Boston, MA United States; 4 Department of Condensed Matter Physics University of Zaragoza Zaragoza Spain

**Keywords:** epidemics, NPIs, nonpharmaceutical intervention, human behavior, Spain, COVID-19, mobility data, epidemic evolution, public health, surveillance, public health intervention, model-based inference

## Abstract

**Background:**

The initial wave of the COVID-19 pandemic placed a tremendous strain on health care systems worldwide. To mitigate the spread of the virus, many countries implemented stringent nonpharmaceutical interventions (NPIs), which significantly altered human behavior both before and after their enactment. Despite these efforts, a precise assessment of the impact and efficacy of these NPIs, as well as the extent of human behavioral changes, remained elusive.

**Objective:**

In this study, we conducted a retrospective analysis of the initial wave of COVID-19 in Spain to better comprehend the influence of NPIs and their interaction with human behavior. Such investigations are vital for devising future mitigation strategies to combat COVID-19 and enhance epidemic preparedness more broadly.

**Methods:**

We used a combination of national and regional retrospective analyses of pandemic incidence alongside large-scale mobility data to assess the impact and timing of government-implemented NPIs in combating COVID-19. Additionally, we compared these findings with a model-based inference of hospitalizations and fatalities. This model-based approach enabled us to construct counterfactual scenarios that gauged the consequences of delayed initiation of epidemic response measures.

**Results:**

Our analysis demonstrated that the pre–national lockdown epidemic response, encompassing regional measures and heightened individual awareness, significantly contributed to reducing the disease burden in Spain. The mobility data indicated that people adjusted their behavior in response to the regional epidemiological situation before the nationwide lockdown was implemented. Counterfactual scenarios suggested that without this early epidemic response, there would have been an estimated 45,400 (95% CI 37,400-58,000) fatalities and 182,600 (95% CI 150,400-233,800) hospitalizations compared to the reported figures of 27,800 fatalities and 107,600 hospitalizations, respectively.

**Conclusions:**

Our findings underscore the significance of self-implemented prevention measures by the population and regional NPIs before the national lockdown in Spain. The study also emphasizes the necessity for prompt and precise data quantification prior to enacting enforced measures. This highlights the critical interplay between NPIs, epidemic progression, and human behavior. This interdependence presents a challenge in predicting the impact of NPIs before they are implemented.

## Introduction

Spain was among the strongest-hit countries worldwide during the first wave, with officially 28,000 fatalities attributed to COVID-19 [1]. In terms of excess deaths, there was an alarming increase of 80% [2], coupled with an attack rate of about 5% [3]. In response to the rapidly rising case numbers, the Spanish authorities implemented stringent containment measures to control the spread of SARS-CoV-2.

The first case in Spain was reported on January 31. The first local transmission was reported on February 26. On March 8, all autonomous communities (Comunidades Autonomas [CCAA]) had reported local transmission. The fast-rising case numbers led the authorities to impose a national lockdown on March 15. Bars, hotels, and restaurants had to close, and individuals were only allowed to leave their homes for work and essential shopping. However, the national lockdown was foregone by various regional measures. On March 10, Madrid and Álava (Basque Country) closed the entire educational system. Other CCAA followed shortly afterward. Madrid and Andalusia closed the gastronomy sector on March 13 and 14, respectively. Additional local measures consisted of the cancellation of festivities and football matches.

The Spanish government declared a state of emergency on March 14, a day before the lockdown took effect. Despite the lockdown being in place, case numbers were still rising toward the end of March [1]. As a result, the national authorities intensified the lockdown between March 28 and April 12, during which all nonessential economic activities came to a halt. The lockdown was then gradually lifted from May 2 onward under the competence of the local authorities (CCAA).

In short, the nonpharmaceutical interventions (NPIs) issued by the national and local authorities, which came along with a strong reduction in mobility, were eventually sufficient to mitigate the daily infections. A thorough analysis of the series of events that shaped the epidemic evolution during the first wave, as performed in various countries [4-10], is essential to design future mitigation strategies of COVID-19 or other emerging respiratory diseases [11,12].

The imposed NPIs and voluntarily practiced social distancing caused a strong reduction in contacts that was reflected by a decrease in mobility. Different studies show that mobility is a robust indicator for the evolution of the epidemic and, hence, the reproduction number [6,9,13-17]. The relationship was most evident during the early phase of the epidemic, when there was no efficient contact tracing in place and only minor use of face masks [16]. Accordingly, a joint analysis of mobility and epidemiological data can provide valuable insight into the evolution of the first wave in Spain.

Moreover, a combined examination provides an opportunity to investigate the factors influencing epidemic trends beyond the implementation of NPIs. Numerous earlier studies that assessed the epidemic's progression assumed sudden shifts in transmission rates as NPIs were implemented [4,18,19]. This method may potentially exaggerate the effects of NPIs, as it does not account for the voluntary behavior modifications of the population that could impact the epidemic's trajectory. By addressing this issue, the integrated analysis presented here offers a more comprehensive perspective on the forces driving the dynamics.

More specifically, in this study, we first evaluate large-scale mobile phone data to show how mobility evolved in the face of rising case numbers and during the lockdown. In particular, we analyze whether there was any change in mobility prior to the introduction of the lockdown. To determine the impact of mobility on the epidemic dynamics, we then contrast the evolution of mobility with epidemiological data, such as case numbers, hospitalizations, and fatalities. More specifically, we blend a direct analysis of the epidemiological data [5,18] and model-based inference [10,19-21]. Furthermore, leveraging the model-based inference, we evaluate the evolution of the underreporting of infections over time. Finally, although most previous studies have focused exclusively on the impact of NPIs, this model-based approach allows us also to build counterfactual scenarios and quantify how the epidemic response that anticipated the lockdown substantially reduced the impact of SARS-CoV-2 in Spain during the first wave.

## Methods

### Mobility Data

The data were provided by the Ministry of Transport, Mobility and Urban Agenda (MITMA) [22]. The raw data stemming from 1 mobile network provider consisted of the anonymized individual trajectories of about 13 million individuals. Beyond the intrinsic limitation of mobile phone data, we assumed that the 13 million individuals would provide a reasonable sample of the Spanish population. By using additional information, such as land usage, sociodemographic indicators, the transport network and the schedule of the public transport, the raw data were transformed into an origin-destination matrix by the MITMA. We directly used the origin-destination matrices and did not have access to the raw data. More details can be found in the methodological note provided by the MITMA [23]. Trips are recorded on the level of municipalities and aggregated on an hourly basis. Additionally, trips are separated into 6 different distance classes. To calculate the mobility reduction on a national or provincial level, we summed up the number of trips and compared it to the corresponding day during the reference period (February 14-20).

### Reconstruction of Exposure Times

We reconstructed the exposure times for different autonomous communities and regions (CCAA) by using a deconvolution process with the symptom onset data. This method allowed us to estimate the time individuals were exposed to the virus before they started exhibiting symptoms, providing a more accurate understanding of the transmission dynamics within each region. By tracing back the exposure times, we can gain valuable insights into the infection patterns and better assess the effectiveness of various interventions and public health measures implemented across the CCAA. This information can then be used to improve and refine epidemic models and guide future decision-making to better control the spread of the virus. More specifically, we used the backprojNP function [24] from the *surveillance* package [25,26] in R. The method, initially proposed by Becker et al [27], infers the expected number of exposures, given the probability mass function of the incubation period, through a maximum likelihood deconvolution approach.

Credible intervals were calculated based on a bootstrap procedure [28]. We fixed the smoothing factor k=6, which corresponds to a centered rolling average of days. The bootstrap procedure made use of 1000 samples (B=1000). The incubation period distribution was fixed as a gamma distribution with mean 5.2 and SD 2.8 days [29]. The time series with symptom onsets was provided by the Centro Nacional de Epidemiología [1].

### Estimation of Rt

From the median incidence, obtained by the reconstruction of the exposure times, we estimated the evolution of Rt using the *EpiEstim* package [30,31]. For the infectivity profile, we chose the generation time estimated by Ganyani et al [32]. To be more precise, we assumed a generation time following a gamma distribution with mean 5.2 (95% CI 3.78-6.78) days and SD 1.72 (95% CI 0.91-3.93) days. This generation time distribution corresponds to the estimation by Ganyani et al [32] for Singapore, while assuming the same incubation period distribution [29] as we did in the reconstruction of the exposure times. We assumed SDs of 1.0 and 1.2 days for the mean and the generation time, respectively. However, we bound the values for the mean and SD by the estimations of Ganyani et al [32]. We fixed a centered rolling average of 7 days. To bootstrap the credible intervals, we took 100 samples of the generation time distribution and considered 100 posteriors for each of these samples (n1=100 and n2=100, respectively).

### Identifying the Linear Segments of Rt

To identify the linear segments, we use the R package *segmented* [33,34]. The method proposed by Muggeo [33,34] implements a maximum likelihood approach using linear predictors. The credible intervals are obtained through bootstrapping. As previously pointed out, we assumed 3 segments of Rt. An initial constant value R1 as the disease was spreading freely in Spain, as well as 2 linear evolving parts corresponding to the decrease in Rt toward the lockdown and the constant decrease observed during the lockdown.

### Model

An alternative method for investigating the evolution of Rt is to perform model-based inference [10,19,20]. By fitting a minimal model to daily fatalities and hospitalizations, we can compare the results with those derived from the reported number of infections and assess whether the early decrease in Rt can be attributed to saturation in testing capacity. This approach is more reliable as fatalities and hospitalizations are less susceptible to fluctuating reporting rates. However, daily fatalities experience significant underreporting. Although the official number of COVID-19 fatalities during the first wave is approximately 28,000, excess deaths amount to around 50,000 [2]. Nonetheless, the reported fatalities and excess deaths follow similar trends, with excess deaths exhibiting a slower decline (Figure S13, Multimedia Appendix 1). Consequently, we considered the reported fatalities to be a sufficiently robust data stream.

We opted for a discrete-time model, informed by empirically derived distributions for the generation time [32], incubation time [29], and time from symptom onset to hospitalization and death [35-37]. The time between symptom onset and hospitalization or death varies significantly by age [35] and region [36]. Since our data were aggregated by age and location, we did not incorporate age stratification or geographical heterogeneity through metapopulations.

Moreover, we assumed an instantaneous reproduction number Rt^M^ with a functional form. The notation Rt^M^ distinguishes this from the reproduction number inferred from reported infections, Rt. The functional form of Rt^M^ was inspired by the Rt inferred from the reported infections.

We divided Rt^M^ into 3 linear segments: the “free” spreading phase before restrictions (constant value R1), the initiation of the epidemic response (linear decrease at time BP), and the lockdown (constant value R2) on March 15. We referred to the intersection between R1 and R2, or the moment Rt^M^ began to decrease, as the breakpoint BP. Along with R1, R2, and the initial number of infected individuals I0, the initiation of the epidemic response remained a free parameter. The assumption that Rt^M^ would reach a constant value R2 upon lockdown implementation was re-evaluated in the sensitivity analysis. This framework allowed us to assess the plausibility of an early decrease around March 5/6, as found in the reported infections.

We decided against using intensive care unit (ICU) admissions as a data stream for our inference. During the first wave, some CCAA reported current occupancy, while others reported new admissions, and many changed their reporting criteria over time. Additionally, only in Madrid and Catalonia, the hardest-hit CCAA, did we observe an earlier peak in the 70-79-year age group compared to younger groups, followed by a rapid decrease in ICU admissions (Figure S14, Multimedia Appendix 1). This strongly suggests that ICU admission criteria were adjusted due to health care system overload. Another indication of this is that hospital admissions and ICU admissions peak on the same day, even though health authorities report a 3-day delay from symptom onset to ICU admission compared to hospital admissions (Figure S15, Multimedia Appendix 1). These factors suggest that ICU admissions data are not a reliable data stream.

Given the form of Rt^M^, the generation time distribution w(t), and the size of the population N, the daily incidence I_t_ on day t evolves as [38]:



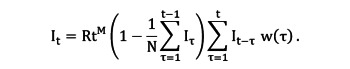



We used the same generation time distribution as that for estimating Rt [32]. From the daily incidence, we propagated the symptom onset as well as the daily fatalities through convolution [14,19]. Given the incubation time distribution P(t), and the distribution of time from symptom onset to hospitalization H(t) and to death D(t), the daily number of individuals developing symptoms S_t_, the daily hospitalizations G_t_, and the daily fatalities F_t_ evolve as:



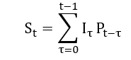





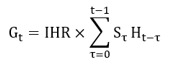





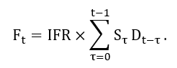



### Model Fitting

We fixed the incubation time distribution as that for the estimation of exposure times [29]. We fixed the infection hospitalization ratio (IHR) and the infection fatality ratio (IFR) by dividing the total number of hospitalizations and fatalities, respectively, by the total number of infected individuals. The latter was fixed through a nationwide seroprevalence study that found an attack rate of 5% by the end of April 2020 [39]. This led to an IHR and an IFR of 4.54% and 1.18%, respectively.

We assumed the times from symptom onset to hospitalization and to death to follow a gamma distribution. Since case line data were not available, we fixed the shape factor as 2.5 and 2.2, respectively, according to Hawryluk et al [35]. However, the health authorities published the median and IQR of these distributions [37]. They reported a median time from symptom onset to hospitalization and death of 6 (IQR 3-9) and 11 (IQR 7-17) days, respectively. We fixed the scale values by performing a least-squares fit with respect to these data. This resulted in a shape and scale parameter for time between symptom onset to hospitalization and death of 2.68 and 5.85, respectively.

We adjusted the model to the daily hospitalizations and fatalities through a Markov chain Monte Carlo (MCMC) approach—to be more precise, through Hamiltonian Monte Carlo [40]. For the log-likelihood, we chose a negative binomial distribution with a dispersion parameter that was left as a free parameter. Motivated by previous findings [13,19], we fixed the prior of R1 as a normal distribution with mean 4.5 (SD 1.0). The prior of R2 was a uniform distribution between 0.4 and 1.0. Similarly, the prior of BP was flat between and days before lockdown. The initial number of infected individuals I0 was uniform between and 5000. We set the prior for the dispersion as a normal distribution with mean 10.0 and SD 5.0. The daily fatalities were not accurately adjusted using directly the distribution received from the least-squares fit. Therefore, we added the scale and shape parameter of the distribution for time between symptom onset and death as a free parameter. We fixed the prior as a normal distribution with a mean as that found from the least-squares fit and SD 0.1. The form of the inferred distribution from symptom onset to death is shown in Figure S20, Multimedia Appendix 1. The model was implemented in Stan [41,42]. We ran 6 chains with 4000 iterations, where 2000 iterations were used for warm-up. Gelman-Rubin convergence statistics [43], that is, potential scale reduction factors, were all smaller than 1.001. Posteriors and trace plots are shown in Figures S21 and S22, Multimedia Appendix 1, respectively.

### Counterfactual Scenarios

A common question when analyzing the pandemic's evolution retrospectively is, What would have happened if we had acted earlier or later? Several studies have examined the impact of an earlier or later lockdown [4,7,20]. These detailed modeling efforts allow us to address this question by considering a shift in the entire epidemic response [7,20]. If the reproduction number is initially constant, as was the case in our analysis, the effect of shifting the epidemic response is entirely determined by R1, that is, the initial doubling time. In other words, if one shifts the epidemic response by the number of days equivalent to the doubling time, the attack rate, hospitalizations, and fatalities double.

Here, however, we focused on the epidemic response that occurred before the lockdown implementation. This encompassed regional measures, individual awareness, nationwide educational system closures, and the lockdown announcement. Essentially, we moved the breakpoint BP by a specific number of days. If the shift surpassed the lockdown date, the BP remained fixed on March 15.

### Sensitivity Analysis

The first part of the sensitivity analysis consisted of relaxing the assumption that the reproduction number would reach a stable value (R2) on March 15, the day the lockdown took effect. To do so, we introduced a second breakpoint BP2 that defined when Rt^M^ would reach R2. For BP2, we chose a flat prior from March 15 onward. The detailed results of the fit are shown in Figures S16-S18, Multimedia Appendix 1. The other estimated parameters, as well as the counterfactual scenarios, were robust. The median of BP2 was found half a day later than March 15, with a credible interval between March 15 and 17. This added plausibility to our assumption that Rt^M^ would reach R2 on March 15. Furthermore, we considered a second generation time distribution taken from Ferretti et al [44]. The generation time corresponded to a Weibull distribution, compared to a gamma distribution in the main text, with mean 5.0 and SD 1.9 days. The results did not substantially change and are presented in Figures S23 and S24, Multimedia Appendix 1.

### Ethical Considerations

The Research Ethics Committee of Rovira i Virgili University deemed this study as exempt from ethics review (URV.F01.04.00 ALTRES-2023-PRD-0001). The mobility data from the mobile phone provider were anonymized and aggregated. Individual traces were not available. No other ethical considerations apply to this work.

## Results

### The Evolution of Mobility

**Figure 1:**
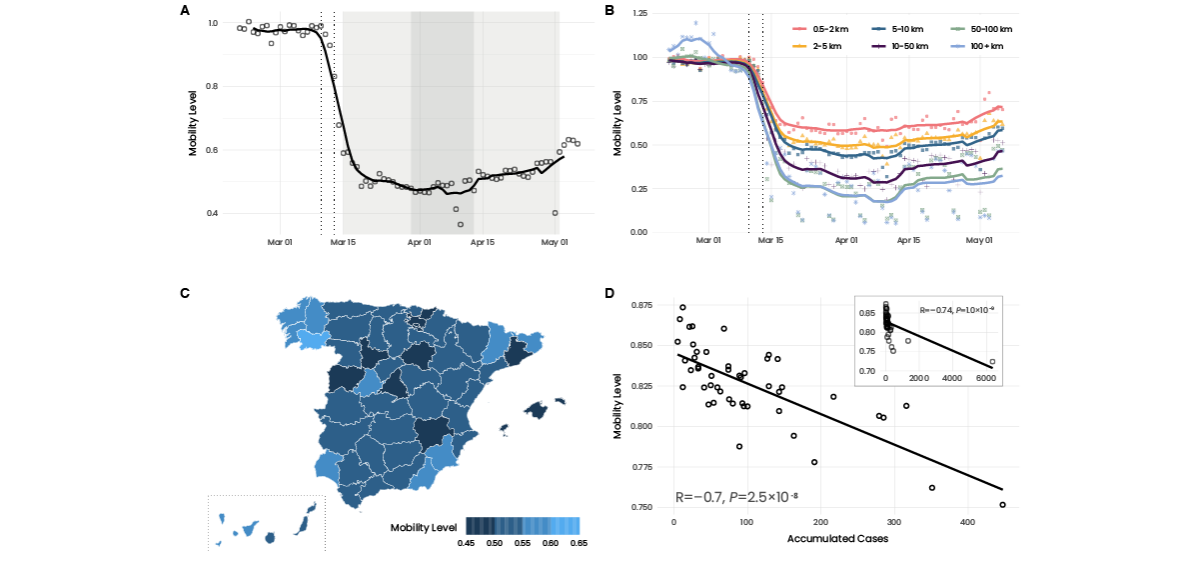
Evolution of mobility during the first wave. (A) Average, nationwide aggregated mobility before and during the lockdown. Dots indicate the data points, while the solid line shows a rolling centered 7-day average. Vertical dashed lines indicate the first NPIs introduced in Spain on March 10 (school closure in Madrid and Basque Country) and the announcement of the lockdown on March 13. The shaded area in light gray indicates lockdowns 1 and 3. In dark gray, we indicated lockdown 2, where, in addition, all nonessential economic activity was shut down. (B) Nationwide aggregated mobility but separating the distances of trips. Dots indicate data points, and the solid lines represent a rolling centered 7-day average. In general, long-distance trips showed a much higher reduction than shorter trips. (C) Mobility level during the lockdown (March 15-May 2) for the total number of trips in the provinces (administrative subdivisions of CCAA) of Spain. Ceuta and Melilla are not shown. Please note that the Canary Islands (islands at the bottom) were moved to be visible. (D) Correlation between the accumulated number of cases until the lockdown and the mobility level. Points represent the provinces of Spain. We excluded the provinces of Madrid and Barcelona since they represent statistical outliers due to their high number of cases. For completeness, the results with these provinces included are shown in Figure S4, Multimedia Appendix 1. The factor R and P denote the Pearson correlation coefficient and the associated *P* value. Similarly, the Spearman correlation coefficient was found to be –0.7 with *P*<.001. CCAA: Comunidades Autonomas; NPI: nonpharmaceutical intervention.

As highlighted previously, mobility is an indicator for the impact of NPIs. Here, we analyzed a data set provided by the MITMA that analyzed the evolution of mobility through anonymized mobile phone data from about 13 million users (see the Methods section for more details). The data indicated that already before the lockdown, there was a substantial reduction in mobility from March 10 onward (Figure 1A). The initiation of this decrease coincided with the first regional NPIs introduced in Madrid and in the Basque country. Furthermore, the early reduction was consistent with an increase in COVID-19–related searches on Google (Figure S1, Multimedia Appendix 1). During the lockdown, mobility was about 50% of the prepandemic level. Mobility increased from the end of the stronger lockdown toward the end of lockdown to about 60%.

Here, we referred to the aggregated, nationwide number of trips. However, the reduction in mobility was heterogeneous. For example, long-distance trips exhibited a much greater reduction than short trips, and long trips were only about 15%-20% of the prepandemic level (Figure 1B). A more detailed overview on the evolution of mobility during the lockdown is provided in Figures S2 and S3, Multimedia Appendix 1. Furthermore, there were regional heterogeneities. If we look at the provincial level (administrative subdivisions of CCAA), the strongest-hit province in terms of infections, Madrid, showed the highest reduction in mobility (Figure 1C). Similarly, highly affected provinces, such as Álava and Barcelona, were among the 5 provinces with the highest mobility reduction.

In fact, we found a pronounced correlation (R=–0.74) between the mobility level and the number of cases (Figure 1D) prior to the lockdown, which then substantially reduced during the lockdown (Figure S5, Multimedia Appendix 1). Interestingly, we observed a stronger correlation with the absolute number of cases than with the population size. The same held true for hospitalizations, ICU admissions, and fatalities (Figure S6, Multimedia Appendix 1).

We did not find regions with increased traffic on the days preceding the lockdown (Figure S7, Multimedia Appendix 1). In addition, the economic level or the fraction of individuals belonging to the working population did not seem to have been factors that determined the mobility level (Figures S8 and S9, Multimedia Appendix 1). However, we observed a general tendency that the reduction in mobility was greater in urban areas than in rural ones (Figures S8 and S9, Multimedia Appendix 1).

### Estimation of Rt

**Figure 2:**
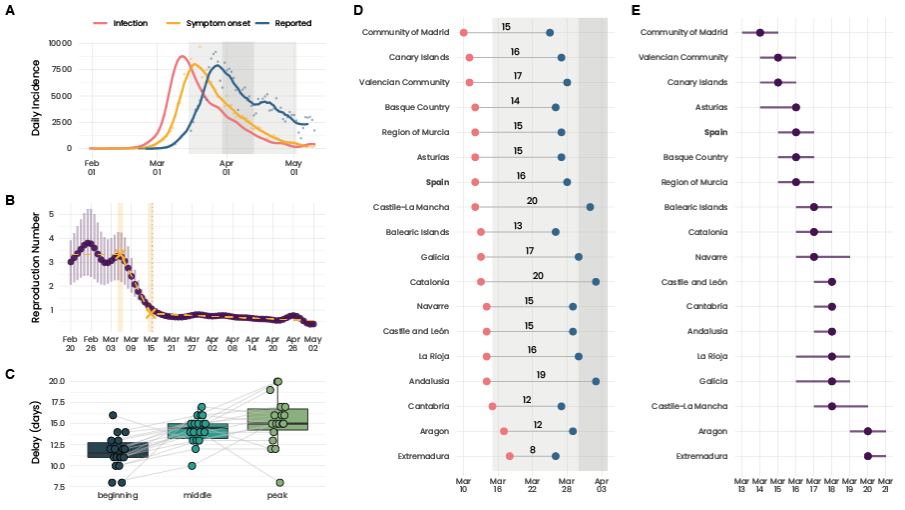
Reconstruction of the exposure times and estimation of Rt. (A) Cases when they were reported (blue), onset of symptoms (yellow), and reconstructed exposure times. Dots indicate data points and the solid line a rolling centered 7-day average. The shaded area for the exposure times represents 95% credible intervals. (B) Estimation of Rt. Vertical bars indicate 95% credible intervals. The dashed yellow line represents the 3 linear segments we identified. The shaded yellow area shows the 95% credible interval for the 2 breakpoints. The 2 breakpoints are between March 5 (95% CI 4-6) and March 15 (95% CI 14-16), where the latter coincides with the implementation of the lockdown. (C) Delay between exposure time and reporting date. The position is defined with respect to the peak. To be more precise, we showed the time difference when both curves reached x% of their peak value. The 3 points (beginning, middle, and peak) correspond to 5%, 50%, and 100%, respectively, of daily infections compared to the peak incidence. Each point corresponds to a CCAA. Gray lines indicate how the delay evolves for each CCAA. We note that the delay steadily increased toward the peak in almost all CCAA. (D) Blue and red dots indicate the day the curves of the reported cases and exposure times reached their respective peaks. The number in between denotes the difference in days between these dates. (E) The day Rt was first below 1 in the different CCAA. Horizontal bars indicate 95% credible intervals. CCAA: Comunidades Autonomas.

Figure 2A shows how the exposure times substantially anticipated the reported infections. The respective curves peaked with a delay of 16 days. In Figure 2B, we observe how the 3 linear segments capture the essential evolution of Rt. Additionally, we see a minimal variation in Rt during the lockdown, and the second breakpoint aligns with the lockdown implementation on March 15. Given that the average incubation time is about 5 days, this indicates a substantial delay from symptom onset until individuals were tested, results were received, and eventual positive cases were reported. We repeated the same analysis for each CCAA (Figure 2D and Figure S10, Multimedia Appendix 1). The delay ranged, among the CCAA, between 8 days in Extremadura and 20 days in Catalonia.

Given the exposure times, we inferred the reproduction number Rt shown in Figure 2B. We estimated that initially, the epidemic spread with a reproduction number of around 3. Rt started to decrease on March 5/6 according to the linear segments we identified. Furthermore, the first time Rt was below 1 was shortly after the beginning of the lockdown: between March 15 and 17. Repeating the same analysis for the regions, we observed that Rt dropped below 1 in all CCAA between March 13 and 21 (see Figure 2E). Please note that the nationwide data are strongly dominated by Madrid due to the high case numbers there. The evolution of the reproduction number in the CCAA is shown in Figure S11, Multimedia Appendix 1.

Although Rt dropping below 1 shortly after the lockdown was expected, the early decrease in Rt that was initiated on March 5/6 was rather surprising. At this point, no NPIs were in place and we did not observe any reduction in mobility. Furthermore, test-trace-isolate was implemented on such a small scale that it seems unlikely to have substantially contributed to the decrease. A possible explanation for this early reduction in Rt could be a saturation in testing capacity.

Looking at the temporal evolution of the delays between infection and reporting dates, we found an indication that the health care system was under increased strain to test and report infected individuals. The delay between infection and reporting continuously increased toward the peak in infections (Figure 2C). Although initially, we found a delay of 12 days, it increased steadily toward the peak of 16 days. Furthermore, this pattern was consistent across almost all CCAA. The only CCAA that showed a reduction in the delay toward the peak were Aragon and Extremadura (Figure S12, Multimedia Appendix 1). Interestingly, these were the CCAA with the lowest delay between the peak in infections and reported cases (Figure 2D) and were the last CCAA to have an Rt below 1 (Figure 2E). This further supports the hypothesis that there was a general overload on the testing facilities that impacted the evolution of Rt, which in turn may have led to the anticipated decrease in Rt.

### Model-Based Inference

**Figure 3:**
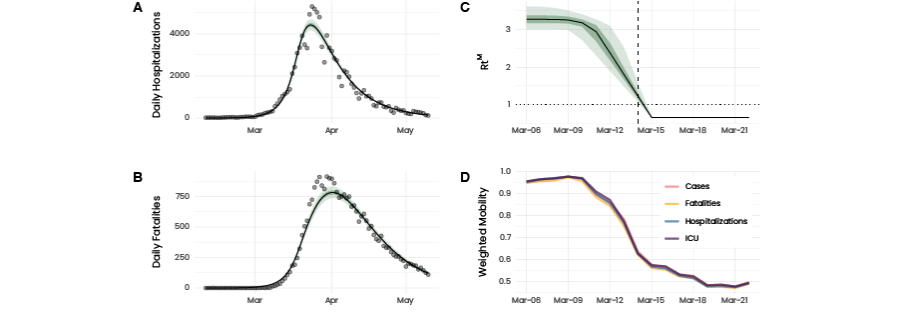
Model adjustment to epidemiological data. (A and B) Model adjustment to the daily hospitalizations and fatalities, respectively. The green and light-green shaded areas represent 50% and 95% credible intervals, respectively. The solid line represents the median. (C) Inferred evolution of Rt^M^. The dashed vertical line indicates the implementation of the lockdown. (D) Aggregated mobility level of the Spanish provinces prior to the lockdown and shortly afterward. Instead of averaging by population size, we averaged by different daily epidemiological indicators to visualize the impact of mobility reduction on epidemic evolution. This is in analogy with the definition of Rt [31]. We observed that mobility and Rt^M^ started to decrease around the same time. ICU: intensive care unit.

Figure 3A,B shows the fit of the minimal epidemic model with respect to the fatalities and hospitalizations, respectively. The adjusted curve for the fatalities peaked slightly later compared to the real data. This could stem from the faster decrease in reported fatalities compared to the excess deaths. Looking at Rt^M^, we found R1=3.27 (95% CI 3.01-3.61) and R2=0.66 (95% CI 0.64-0.67). Both R1 and R2 were consistent with the estimation from the reported cases, Rt. Given the generation time we used here, this resulted in a doubling time of 2.55 days (95% CI 2.32-2.78) during the free phase and a half lifetime of 9.11 (95% CI 8.72-9.56) days after the lockdown. The small doubling time suggests how the considerable delay between infection and reporting dates may have contributed to a substantial discrepancy between the actual epidemiological situation and the one that we could infer from the data. However, the low value of R2, together with the half-life time, shows the efficiency of the lockdown during the first wave and how it contributed to the low case numbers during the early summer months.

Let us turn now to the initiation of the epidemic response. The model adjustments yielded the initiation of the decrease in Rt^M^ on March 10 (95% CI 8-12), contrasting with the decrease in Rt on March 5/6. This suggests that the early decrease was due to an overload in the health care system, which eventually impeded testing infected individuals. The initiation of the decrease in Rt^M^ was consistent with the reduction in mobility. In Figure 3C,D, we contrast the reduction in mobility with Rt^M^. Note that we weighted the mobility reduction by various daily epidemiological indicators—reported cases, fatalities, hospitalizations, and ICU admission—in the different provinces rather than by population. Hence, we accurately reflected the impact of mobility reduction on the evolution of the epidemic. Note that there was an additional, even though less rapid, decrease in mobility from March 15 onward. However, results from the sensitivity analysis (Figures S16-S18, Multimedia Appendix 1) suggest that the reproduction number reached a stable value timely after the lockdown. In this sense, the impact of the additional decrease in mobility on the epidemic seems not to have been substantial.

The eventual saturation of testing capacity also had consequences for ascertainment, that is, the fraction of cases that were detected compared to the total number of infections. We assumed our model output to be the total number of infections and compared it to the number of infections (exposure times) that we inferred previously. We noted that initially, ascertainment was only around 5% and subsequently increased. However, it then substantially dropped around May 5/6 before eventually starting to increase again. This substantial increase was consistent with the pronounced expansion in testing capacity in the middle of April (Figure S19, Multimedia Appendix 1) that enabled the detection of around 15% of the infections.

### Counterfactual Scenarios

**Figure 4:**
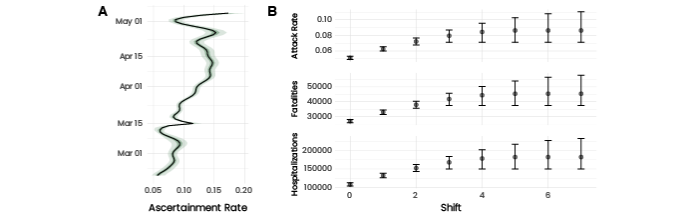
Ascertainment and counterfactual scenarios. (A) Evolution of ascertainment in time. Ascertainment is defined as the ratio between the reported cases and the incidence from the adjusted model. We compared the date of infection (exposure time) instead of the reporting date. We noted a sudden decrease from March 5/6 toward the lockdown. Later, the testing capacity substantially increased, which increased ascertainment (Figure S19, Multimedia Appendix 1). (B) Counterfactual scenarios for the attack rate, death toll, and total number of hospitalizations. The counterfactual scenarios consist of shifting the breakpoint BP by x days. If the BP exceeded March 15 (ie, lockdown), no further shift was applied. We noted how the response before the implementation of the lockdown substantially contributed to limiting the impact of the epidemic.

The results of the counterfactual scenarios, shown in Figure 4B-D, indicate that the absence of an epidemic response before the lockdown would have resulted in an attack rate of 8.6% (95% CI 7.1-11.0), which is more than 50% higher than the actual value. The higher attack rate would then have resulted in 45,400 (95% CI 37,400-58,000) fatalities and 182,600 (95% CI 150,400-233,800) hospitalizations compared to 27,800 fatalities and 107,600 hospitalizations, respectively, that were reported. In other words, the results suggest that the pandemic response before the lockdown contributed substantially to limiting the impact of SARS-CoV-2 during the first wave in Spain.

## Discussion

### Principal Findings

Our analysis of large-scale mobility data revealed a significant reduction in mobility initiated before the lockdown. Additionally, we discovered a strong correlation between the decrease in mobility before the lockdown and the number of reported cases across Spanish provinces. Although the direct analysis of reported infections suggested a reduction in the reproduction number preceding the decrease in mobility, the model-based inference using hospital admissions and deaths primarily attributes this to a declining reporting rate as the epidemic progressed. The model-based inference indicates a simultaneous decrease in mobility and the reproduction number. According to our counterfactual scenarios, we estimate that the reduction in the reproduction number before the lockdown reduced the attack rate, hospital admissions, and deaths by over 30%.

The direct analysis of reported cases revealed a constant increase in the delay between exposure and notification as the epidemic peaked. An average delay of 16 days highlights the late testing of symptomatic individuals, a significant test turnaround time, and considerable notification delays. Studies have reported similar delays in other countries [17,20]. Such delays obstruct accurate evaluations of the epidemiological situation and hinder effective epidemic response management [45].

The increasing reporting delay also suggests a saturation in testing capacity. We hypothesized that the reduction in mobility before the decrease in Rt resulted from a worsening reporting rate. Our model-based approach supports this hypothesis, indicating an epidemic evolution consistent with the reduction in mobility, as found in other studies [15-17,46]. Moreover, ICU capacities seemed to have reached their limits. Specifically, ICU admissions among 70-79-year-olds peaked earlier than for younger age groups in the hardest-hit provinces, Madrid and Barcelona, indicating a change in admission criteria. These factors illustrate the immense pressure the Spanish health care system faced during the first wave.

The decrease in mobility began on March 9/10, 5-6 days before lockdown implementation. Linka et al [17] observed a similar decrease in mobility before lockdown introductions in other Western European countries. Identifying the exact cause of the early mobility decrease is challenging, as various factors are likely to have contributed to the decline. Worsening situations in many countries, reported in the media [47] and further disseminated through social networks [48], might have increased public awareness. In line with this increased awareness, the usage of SARS-CoV-2–related hashtags in Spain surged from March 9 onward [49]. We also found a significant correlation between mobility levels and the number of cases at the provincial level, suggesting the presence of risk-based individual awareness [50]. Other studies have identified risk-based awareness for SARS-CoV-2 [51,52], as well as for HIV [53] and measles [54].

The correlation between mobility and reported cases was especially high before lockdown implementation, as contact reduction mainly occurred voluntarily (Figure S5, Multimedia Appendix 1). However, the first autonomous communities announced educational system closure on March 9, with many others following suit. These factors are interconnected, as public awareness of COVID-19 influences voluntary social distancing, and individuals' attitudes toward the disease also impact authorities' decisions. Moreover, lockdown announcements can have similar effects as their implementations [55]. Our results should not be interpreted as lockdowns and NPIs being generally ineffective. Various studies have emphasized the impact of NPIs [18,56]. Our findings, however, highlight the complex interplay between risk-based and policy-induced behavioral changes and challenge a mechanistic understanding of NPIs, where any reduction in the reproduction number is solely attributed to policy [10,19]. In summary, studies analyzing the impact of public health policies should also account for voluntary behavioral changes beyond NPIs.

At the beginning of the model dynamics on February 10, we estimated around 1100 infectious individuals. In contrast, the first case of local transmission in mainland Spain was reported on February 26. This discrepancy underscores how SARS-CoV-2 initially spread silently through the population. This observation aligns with the excess deaths attributed to influenza in February 2020 in Catalonia [57]. In line with this, the model consistently predicts more fatalities at the beginning of the epidemic than were reported.

Our counterfactual study demonstrates that the early decrease in mobility significantly contributed to mitigating the impact of the first wave in Spain. Our results suggest that if the epidemic response had started with the lockdown implementation on March 15, the attack rate would have been 8.6%, resulting in 180,000 hospitalizations and 45,000 fatalities, an increase of 60%-70% in these indicators. This finding indicates that in addition to the national lockdown, the combination of awareness and regional measures helped slow the spread of SARS-CoV-2 in Spain. Shifting the entire epidemic response, including the lockdown, would have led to twice/half as many infected individuals for every 2.5 days later/earlier. This emphasizes the importance of timely containment efforts in managing emerging epidemics [7,19,20,58].

### Limitations

Several aspects of our analysis could be improved if more detailed data were available. For instance, the epidemiological model could be age-stratified if relevant data were available for Spain regarding symptom onset to hospitalization and death [36]. The same applies to geographical heterogeneity. If case line data were available at the regional level, autonomous communities could be treated separately [35]. In the absence of such data, age or location stratification is not feasible. Access to data that are not publicly available, unlike in other countries [4], would enable a more detailed and comprehensive analysis of the 2020 SARS-CoV-2 epidemic in Spain [11,12].

### Conclusion

Summarizing, this study emphasizes that the behavioral response to an epidemic is multifaceted, driven by both voluntary decision-making and health authorities' policies. Although our results indicate that the reproduction number reached its lowest point during the lockdown in Spain, they also suggest a decrease before the implementation of any containment policies. Disentangling voluntary from policy-induced behavioral changes remains a future challenge, as this interplay involves individual psychology, societal dynamics, and pathogen spread [59]. However, understanding this complex interplay is crucial for designing better health policies and accurately assessing the need for public health interventions.

## Appendix

Multimedia Appendix 1 Complementary figures.

## Abbreviations

CCAA: Comunidades Autonomas
ICU: intensive care unit
IFR: infection fatality ratio
IHR: infection hospitalization ratio
MITMA: Ministry of Transport, Mobility and Urban Agenda
NPI: nonpharmaceutical intervention
