# Supplementary material for: Joint Analysis of the Epidemic Evolution and Human Mobility During the First Wave of COVID-19 in Spain: Retrospective Study
Source: JMIR Public Health Surveill. 2023 May 22;9:e40514. doi: 10.2196/40514 (PMC10208305; doi:10.2196/40514)
Supplement: Multimedia Appendix 1 [file publichealth_v9i1e40514_app1.pdf]

Retrospective study of the first wave of COVID-19 in Spain: A joint analysis of the epidemic evolution and human mobility

Supplementary Information

Benjamin Steinegger[a], Clara Granell[a], Giacomo Rapisardi[b], Sergio Gómez[a], Joan T. Matamalas[c], David Soriano-Paños[d,e], Jesús Gómez-Gardeñes[d,e], Alex Arenas[a,*]

[a] Departament d'Enginyeria Informàtica i Matemàtiques, Universitat Rovira i Virgili, 43007 Tarragona, Spain
[b] Barcelona Supercomputing Center (BSC)
[c] Center for Interdisciplinary Cardiovascular Sciences, Cardiovascular Division, Department of Medicine, Brigham and Women's Hospital, Harvard Medical School, Boston, MA 02115, USA
[d] Department of Condensed Matter Physics, University of Zaragoza, 50009 Zaragoza, Spain
[e] GOTHAM Lab-BIFI, University of Zaragoza, 50018 Zaragoza, Spain

[*] Corresponding author. E-mail: benjaminfranzjosef.steinegger@urv.cat,alexandre.arenas@urv.cat

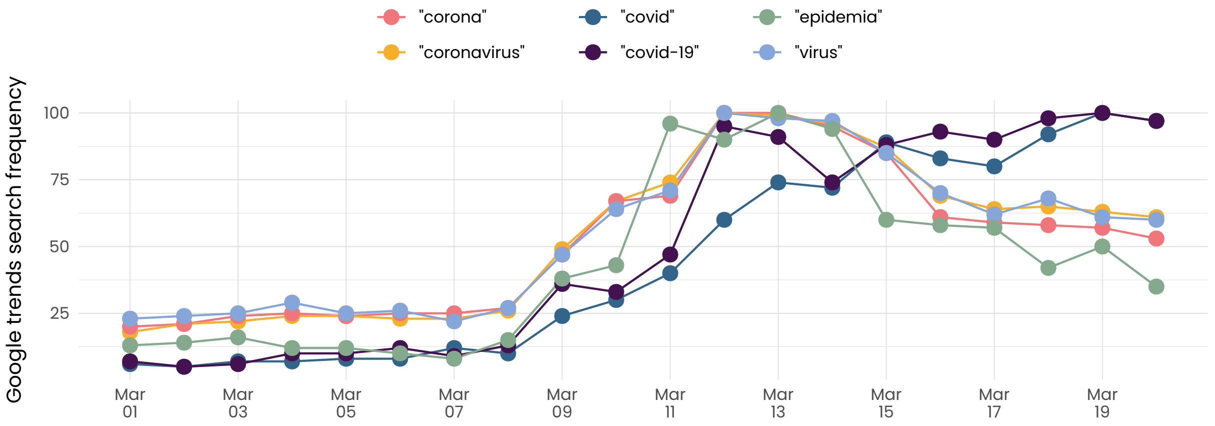

Figure S1: Frequency of different COVID-19 related search queries according to Google Trends. Note that Google Trends normalizes the frequency of queries between 0 and 100 during the considered period. All key words see a sharp increase from March 9 onward. On the same day, the Community of Madrid [**?**] and the Basque Country [**?**] announced the closure of educational centers. These closures correspond to the first NPIs introduced in Spain.

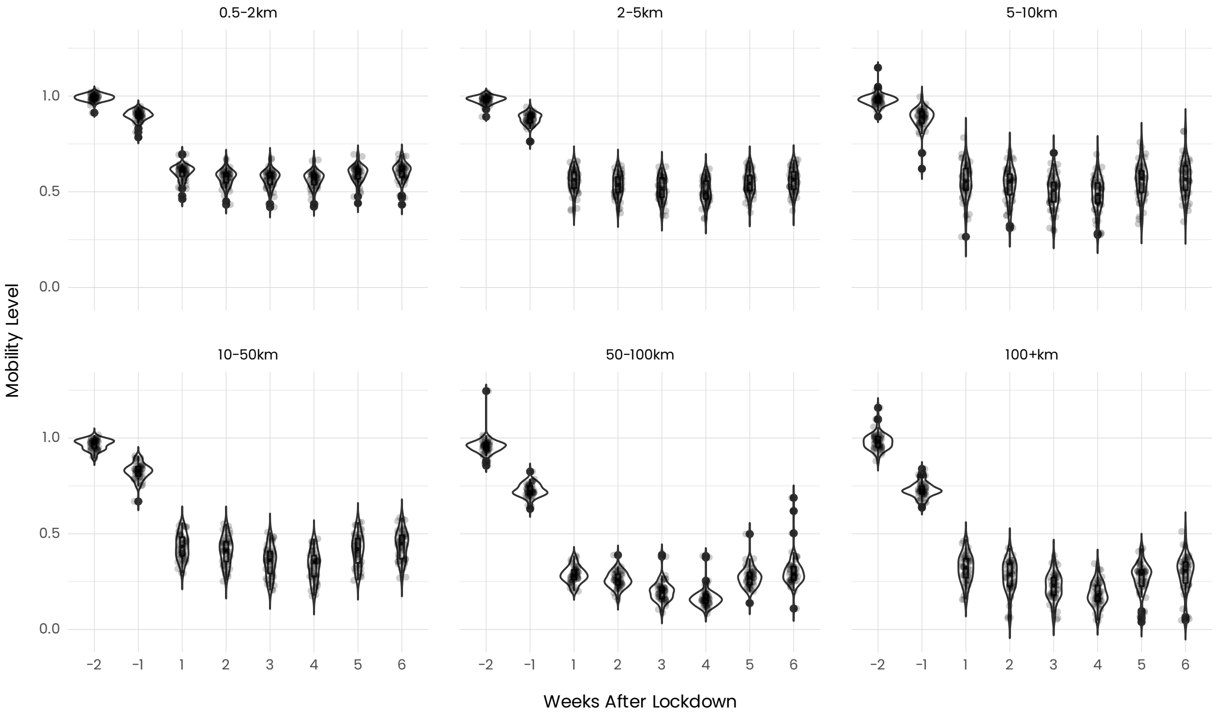

Figure S2: Violin plot of the mobility level [?] during different weeks and separated with respect to the distance of trips. Each data point corresponds to a province in Spain. To have a reference, the weeks -1/1 correspond to the weeks March 9–15/16–22. We observe that the reinforcement of lockdown (weeks 3/4) leads to an additional decrease in mobility, mainly for distances above 10km.

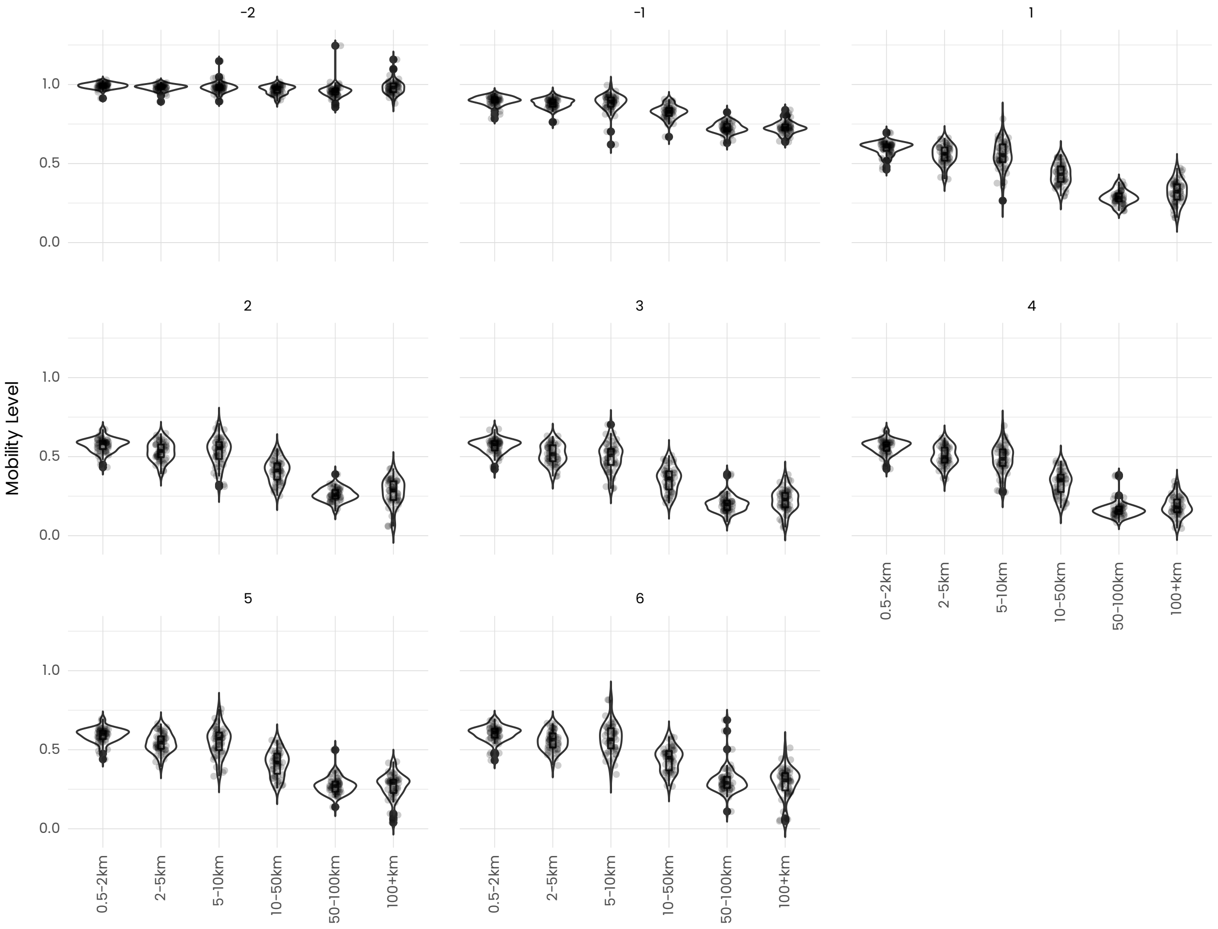

Figure S3: Violin plot of the mobility level [?] with respect to distances of trips and separated for different weeks. Each data point corresponds to a province in Spain. To have a reference, the weeks -1/1 correspond to the weeks March 9–15/16–22. Longer distances see a stronger reduction in mobility. Similarly, we observe that before lockdown (week -1), long trips were reduced by about 25%. This shows that individuals reduced non-essential trips before lockdown, indicating a general awareness of the population.

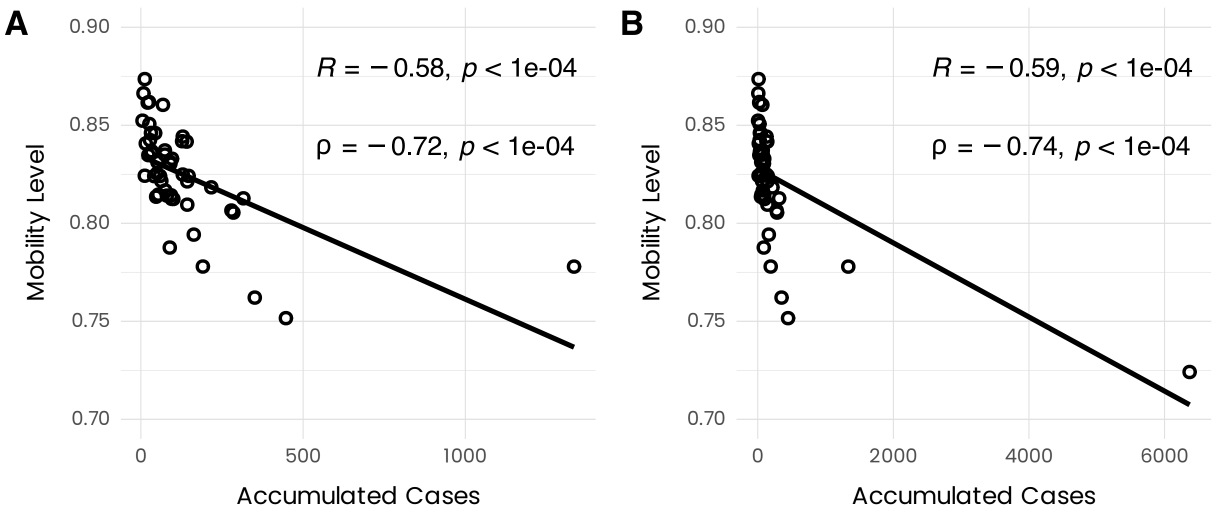

Figure S4: Correlation between the mobility level [**?**] and accumulated number of reported cases. The correlation was calculated through the Pearson R and Spearman ρ correlation coefficient. In contrast to Fig. 1D, the panel A includes the province of Barcelona. The panel B includes Barcelona and Madrid. The inclusion of Barcelona and Madrid suggests a non linear relationship between the mobility level and the number of cases. Accordingly, the Spearman coefficient varies less as Madrid and Barcelona are included.

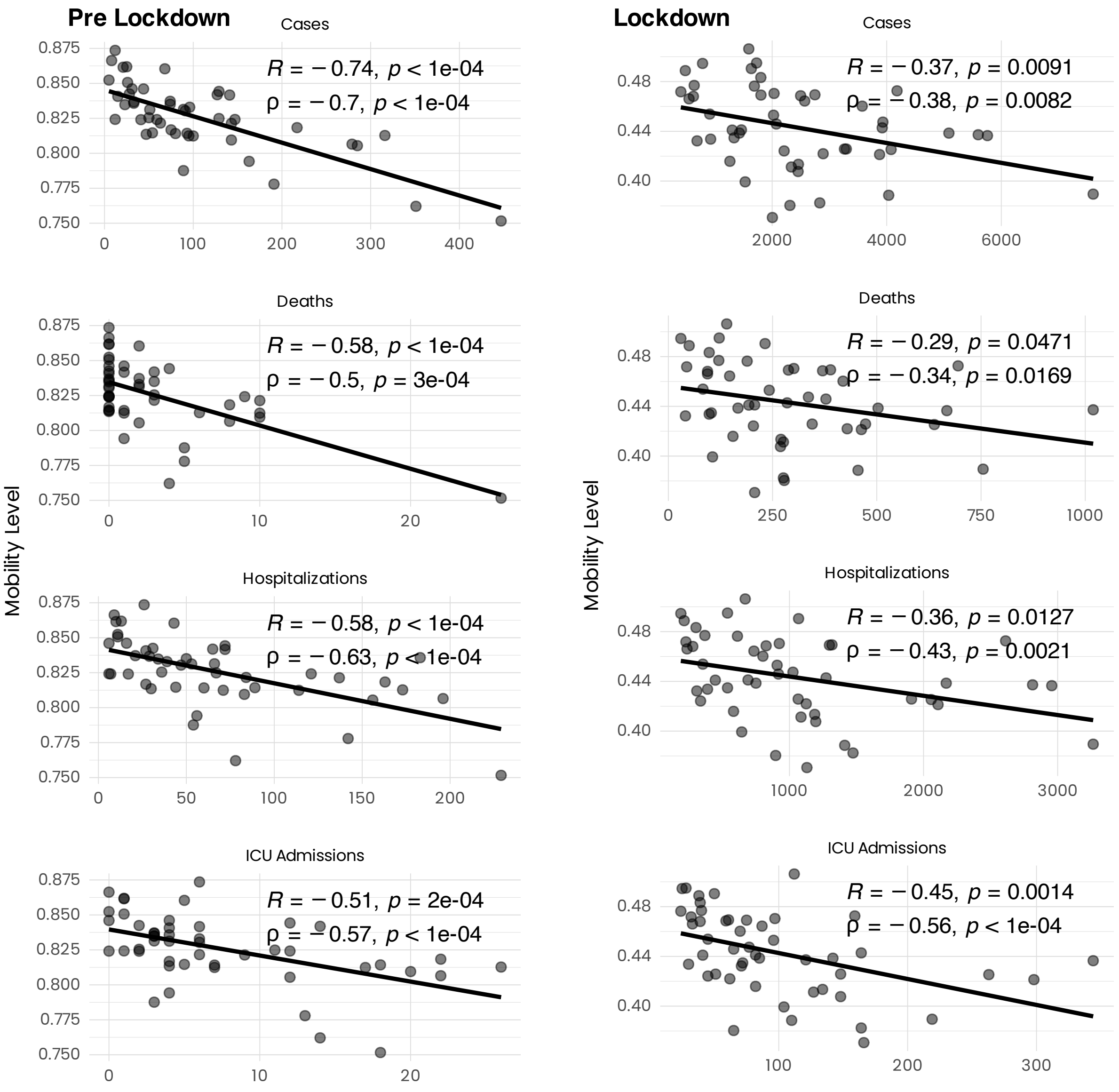

Figure S5: Correlation between the mobility level [?] and accumulated number of reported cases, deaths, hospitalizations and ICU admissions [?]. Each data point corresponds to a province. The correlation was calculated through the Pearson R and Spearman ρ correlation coefficient. We excluded Ceuta and Melilla due to their size. Barcelona and Madrid were excluded since they are statistical outliers. The left side focuses on the week previous to lockdown from March 9–15. The right side aggregates the period of the lockdown from March 15 to May 5. We observe that correlation between cases and mobility is reduced during lockdown compared to pre Lockdown. This reduction may be explained due to the stay-at-home order that reduced the effect of individual voluntary behavior.

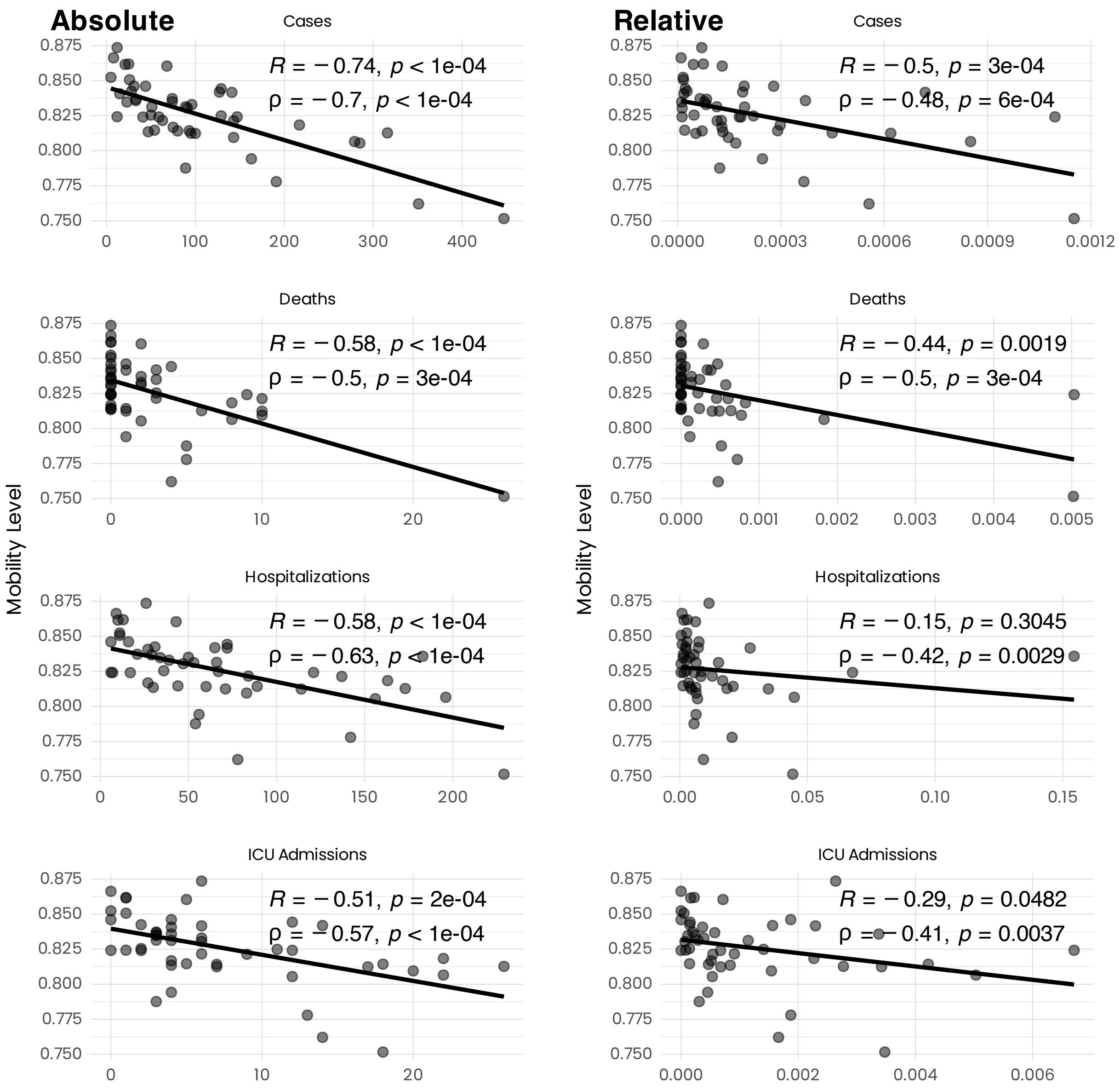

Figure S6: Correlation between the mobility level [?] and accumulated number of reported cases, deaths, hospitalizations and ICU admissions [?] during the week previous to lockdown from March 9–15. Each data point corresponds to a province. The correlation was calculated through the Pearson R and Spearman ρ correlation coefficient. We excluded Ceuta and Melilla due to their size. Barcelona and Madrid were excluded since they are statistical outliers. The left side takes absolute values of the epidemiological indicators, while the right normalizes them with respect to the population. Interestingly, we find stronger correlation regarding all indicators for absolute rather than relative case numbers.

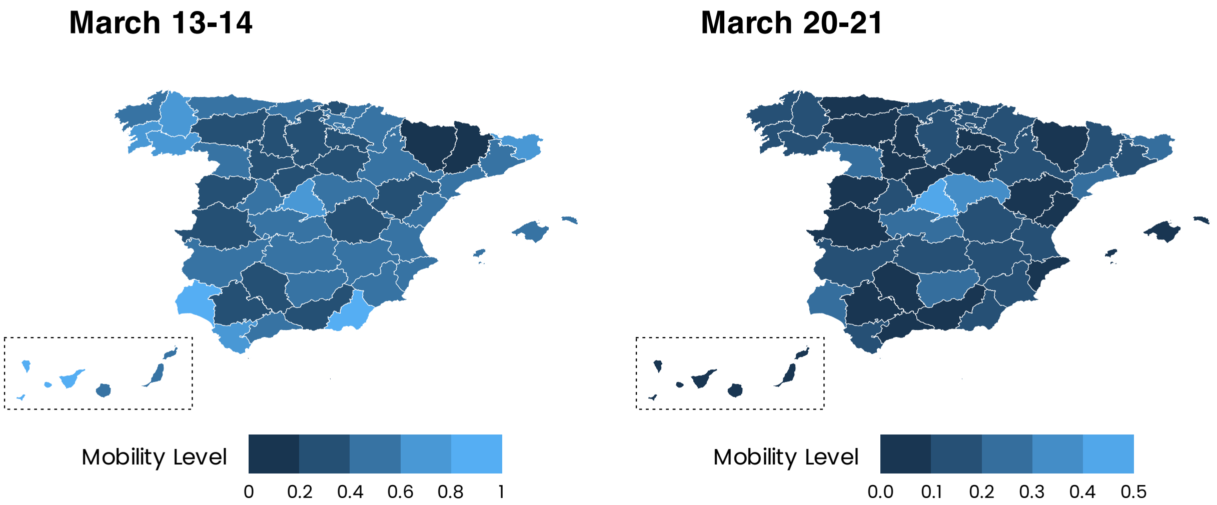

Figure S7: Mobility level [?] of trips that started in the province of Madrid towards the other provinces in Spain between March 13–14 and 20–21. The mobility level is defined with respect to the reference days on February 14 and 15. Observed traffic and newspapers suggested a huge exodus from Madrid outwards during March 20–21 [?]. We do not observe a mobility level that would justify such hypothesis. In contrast to the example of France [?], we do not observe increased mobility levels from the capital to the periphery before lockdown.

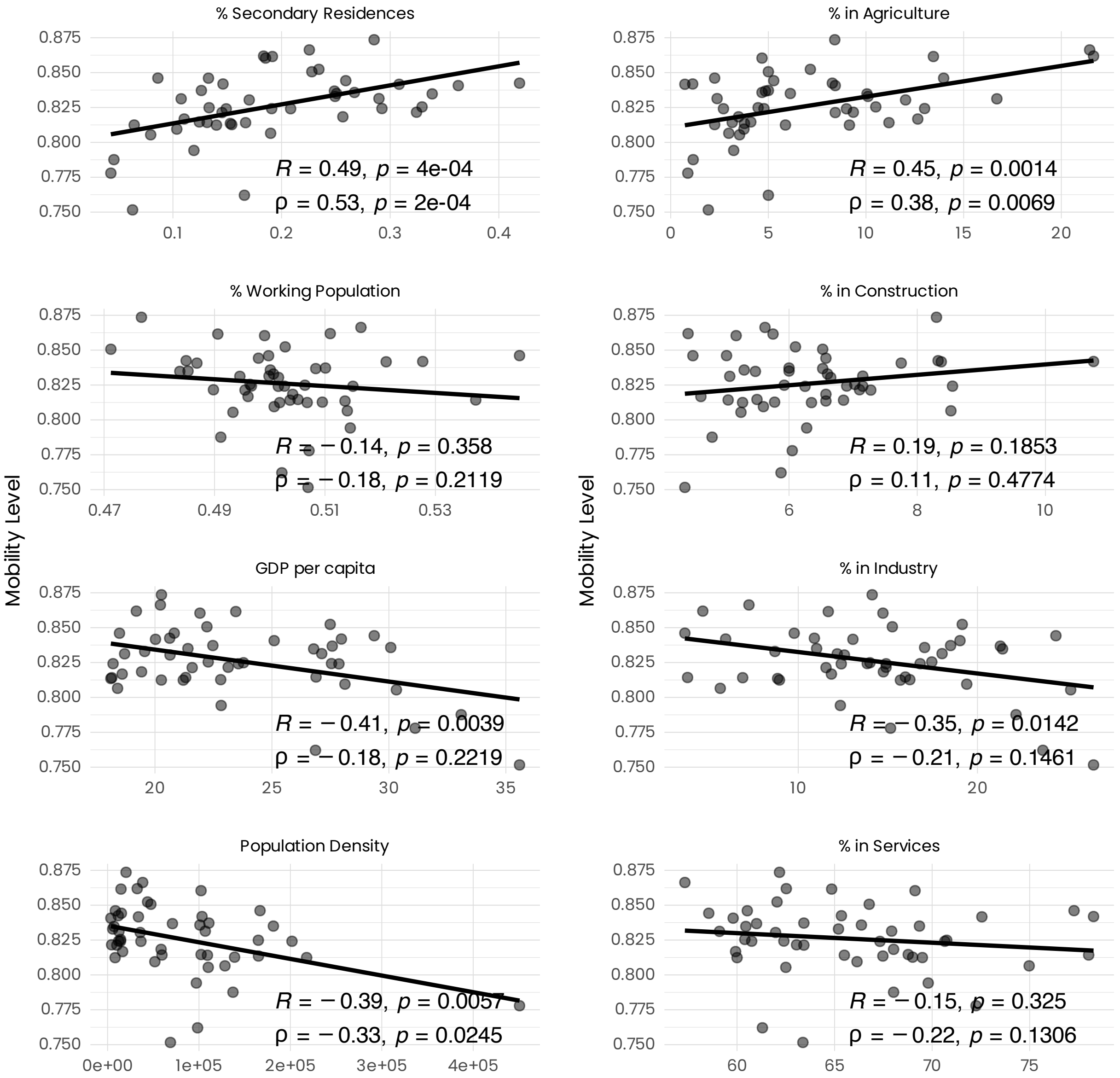

Figure S8: Correlation between the mobility level [?] and different indicators during the week previous to lockdown from March 9–15. Each data point corresponds to a province. The correlation was calculated through the Pearson R and Spearman ρ correlation coefficient. We exclude Ceuta and Melilla due to their small size. We do not find very pronounced correlation with any indicator. The right side analyses in which sectors (Agriculture, Construction, Industry, Services) the population works [?]. The left hand side focuses on the fraction of secondary residences [?], the proportion of the working population (25–65 years old) [?], GDP per capita [?] and population density [?]. The main tendency one can observe is that in rural regions (secondary residences, population density and agriculture) mobility reduction is less pronounced than in urban areas.

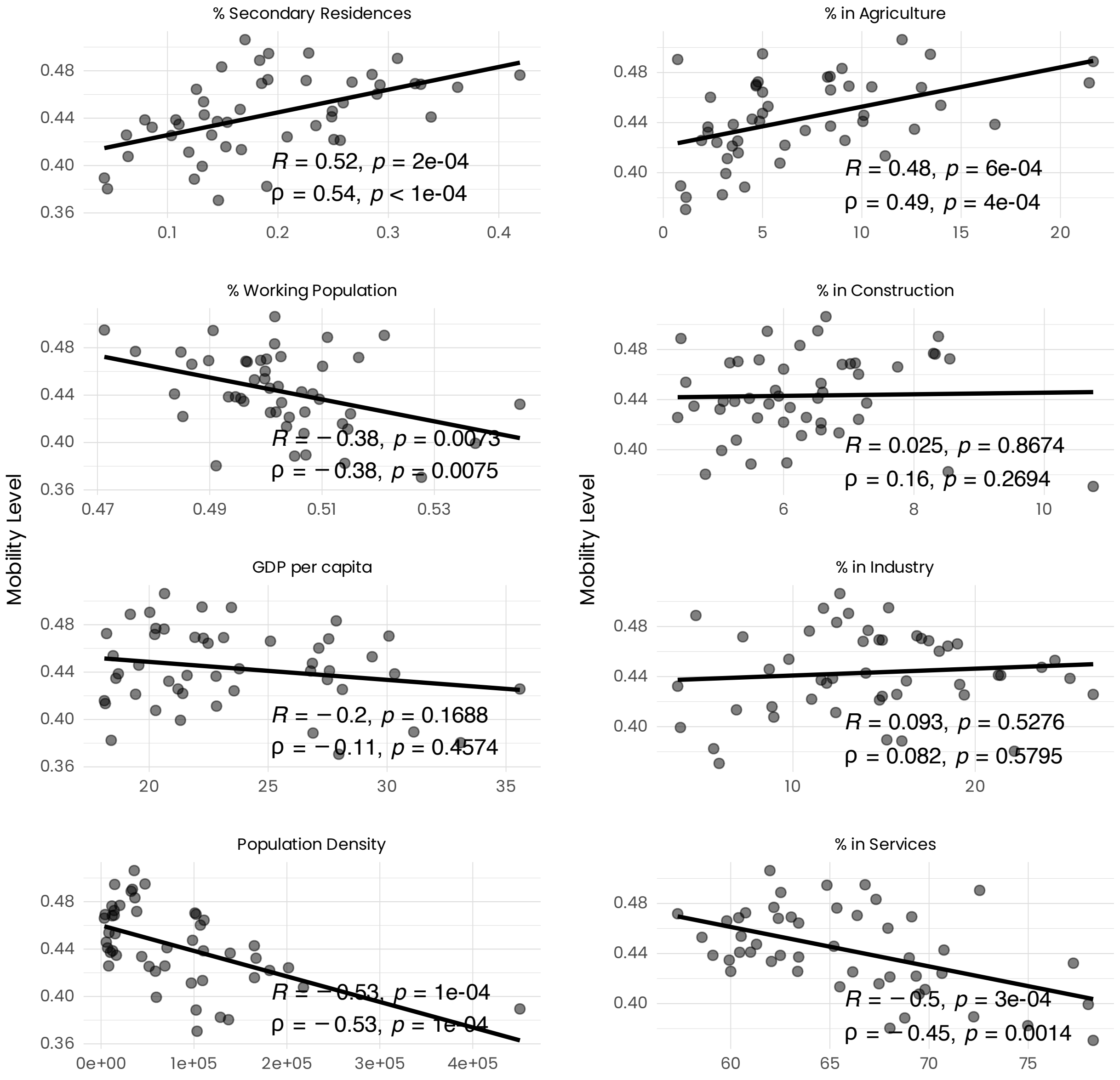

Figure S9: Correlation between the mobility level and different indicators during lockdown from March 15 to May 5. Each data point corresponds to a province. The correlation was calculated through the Pearson R and Spearman ρ correlation coefficient. We excluded Ceuta and Melilla due to their size. We do not find very pronounced correlation with any indicator. Similarly, as previous to lockdown (see Fig. S8), the main tendency one can observe is that in rural regions mobility reduction is less pronounced than in more urban areas.

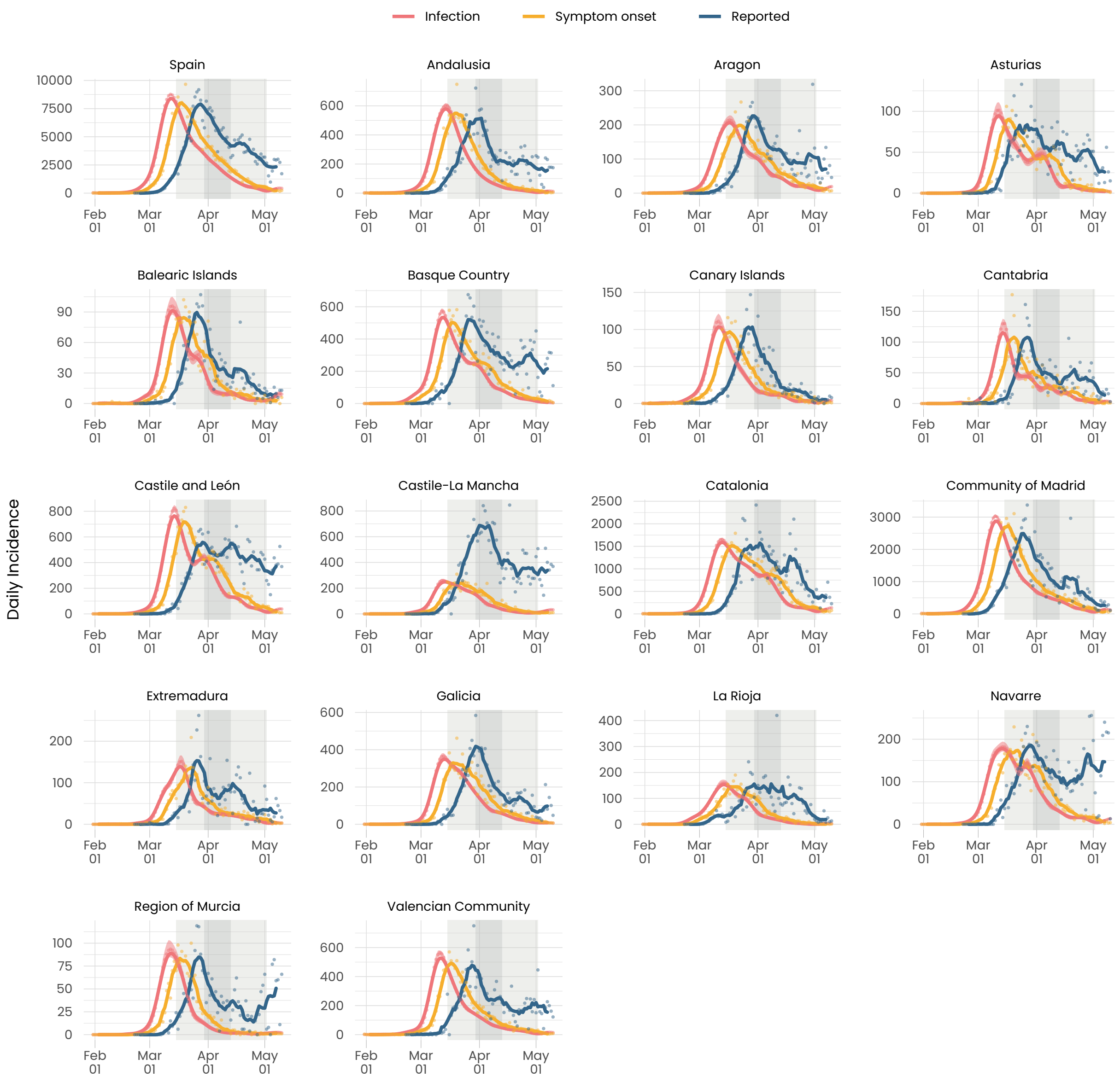

Figure S10: Time series for reported cases in blue and symptom onset in yellow for Spain and the CCAA. Solid lines indicate a centered, seven day rolling average. In red we show the reconstructed exposure times. The shaded area in light grey indicates the lockdowns 1 and 3. In dark grey, we indicate lockdown 2 where, in addition, all non-essential economic activity was shut down.

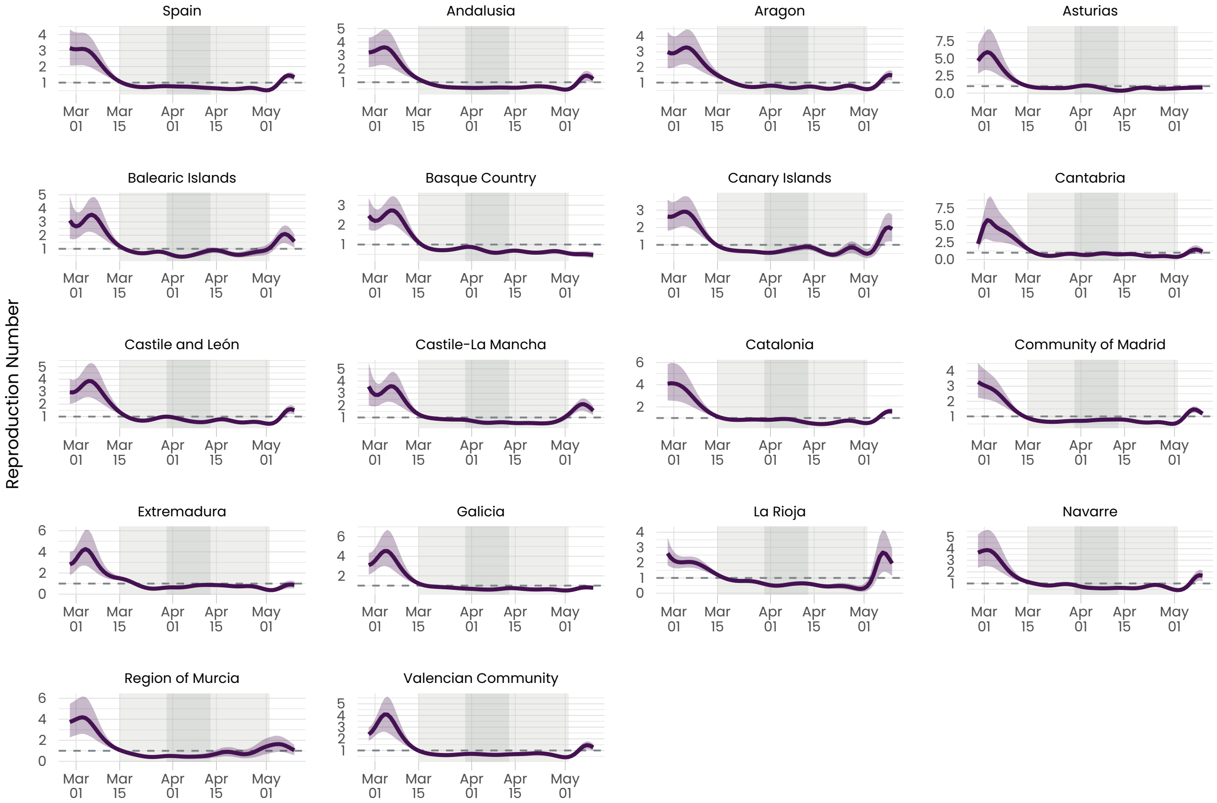

Figure S11: Time series for the inferred Rts for Spain and the CCAA. Solid lines indicate a centred, seven day rolling average. In red we show the reconstructed exposure times. The shaded area in light grey indicates the lockdowns 1 and 3. In dark grey, we indicate lockdown 2 where, in addition, all non-essential economic activity was shut down.

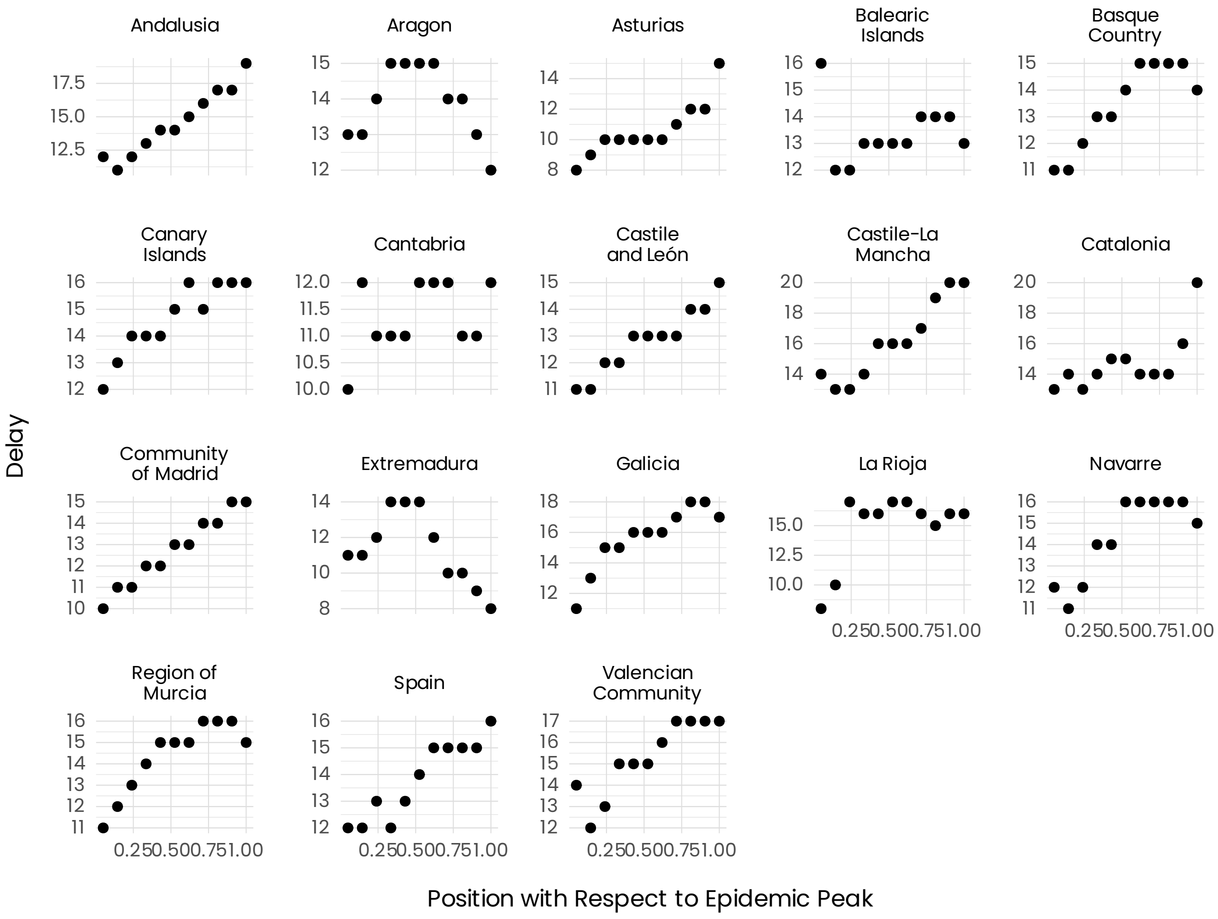

Figure S12: Delay between the exposure time and the reporting date. The position is defined with respect to the peak. To be more precise, we show the time difference when both curves reached x% of their peak value. Each point corresponds to a CCAA. We note that the delay steadily increases towards the peak in almost all CCAA. Exceptions are Aragon and Extremadura.

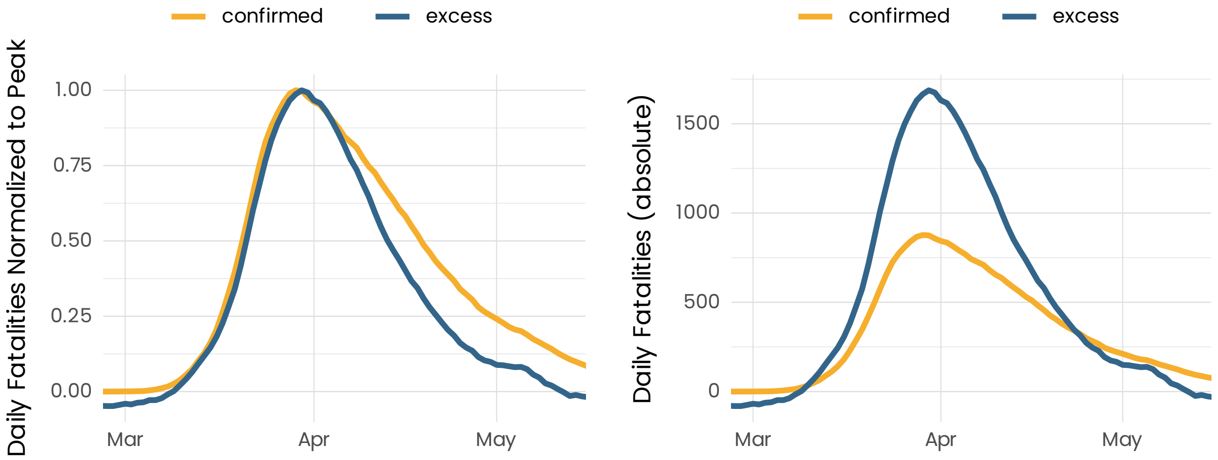

Figure S13: Evolution of the daily fatalities. The left panel shows the evolution normalized with respect to the peak value, while the right panel shows the fatalities in absolute terms. We applied a 7 day centered rolling average to the data. We show reported deaths [**?**] and excess deaths [**?**]. While the rise in fatalities is almost equivalent between the two curves, the daily excess deaths decrease much faster than the reported fatalities.

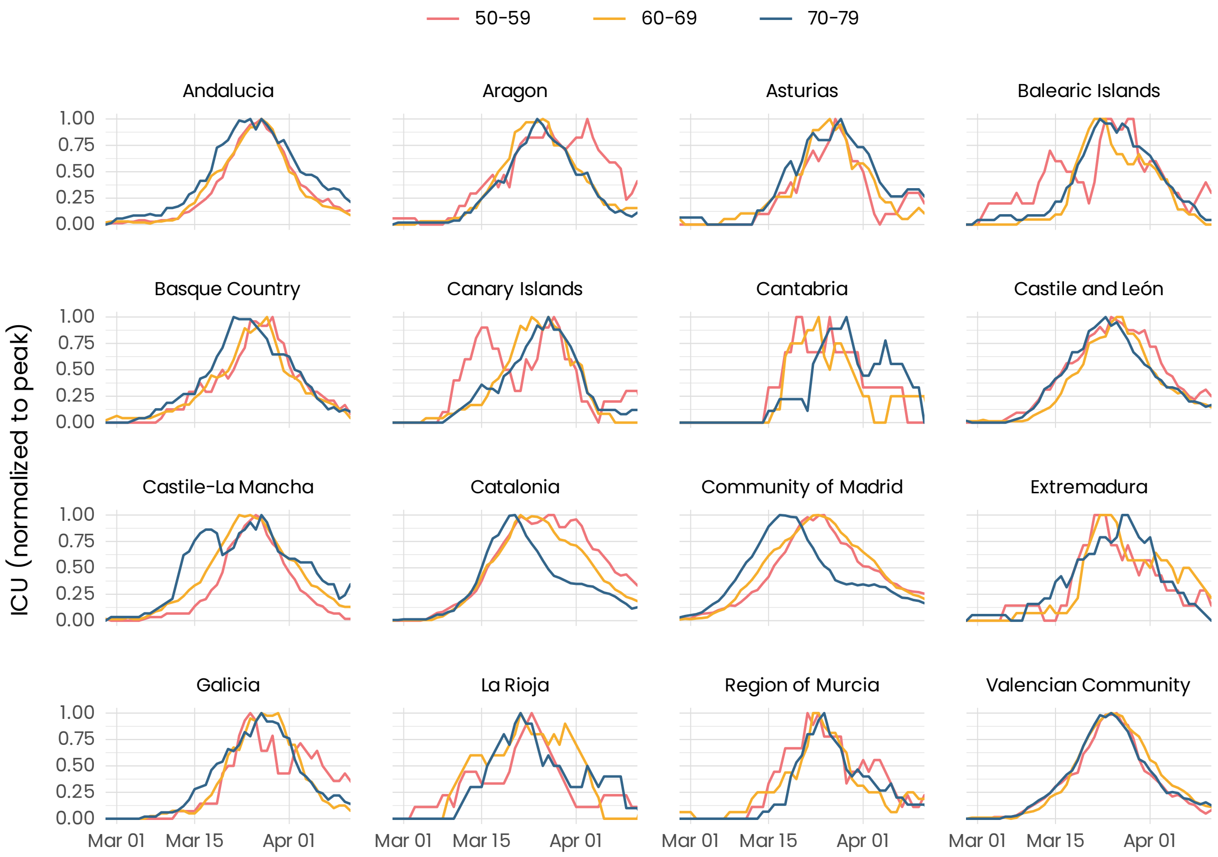

Figure S14: Seven day centered rolling average for the evolution of ICU admission for different age groups separated with respect to CCAA [**?**]. We excluded CCAA were numbers were too low to exhibit a robust evolution. Admission were normalized with respect to their peak value. We observe that in the strongest hit CCAA, Catalonia and Madrid, the admission of individuals in the age group 70–79 peak earlier and decrease much faster. In contrast, in most other CCAA the evolution of admission among different age groups is very similar. It is very probable that changing admission criteria lead to this fast decrease. We could not find any other explanation for this discrepancy. Among other, this inconsistency let us to not include ICU admission as a data stream for our model based inference.

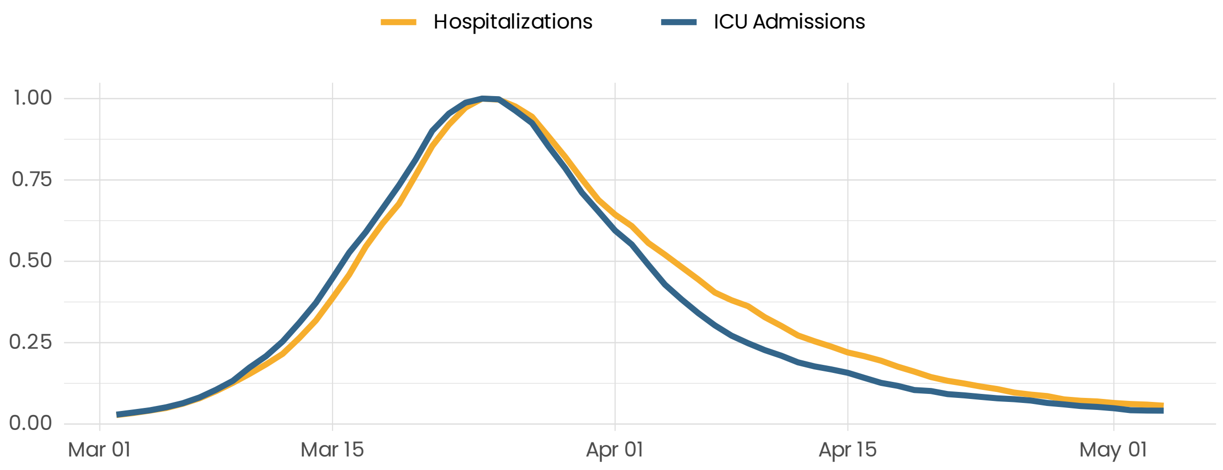

Figure S15: Evolution of daily hospital and ICU admissions [?]. Both curves are normalized with respect to their peak value. Surprisingly, both curves reach their peak on the same day and ICU admissions decrease faster, despite official data indicating a 3 day longer interval between symptom onset and admission for ICUs [?]. This discrepancy further highlights the difficulty to use ICU admissions as a data stream. In line with the sudden decrease in ICU admission for the age group 70-79, a possible explanation is that admission criteria for ICU admission become more stringent over time.

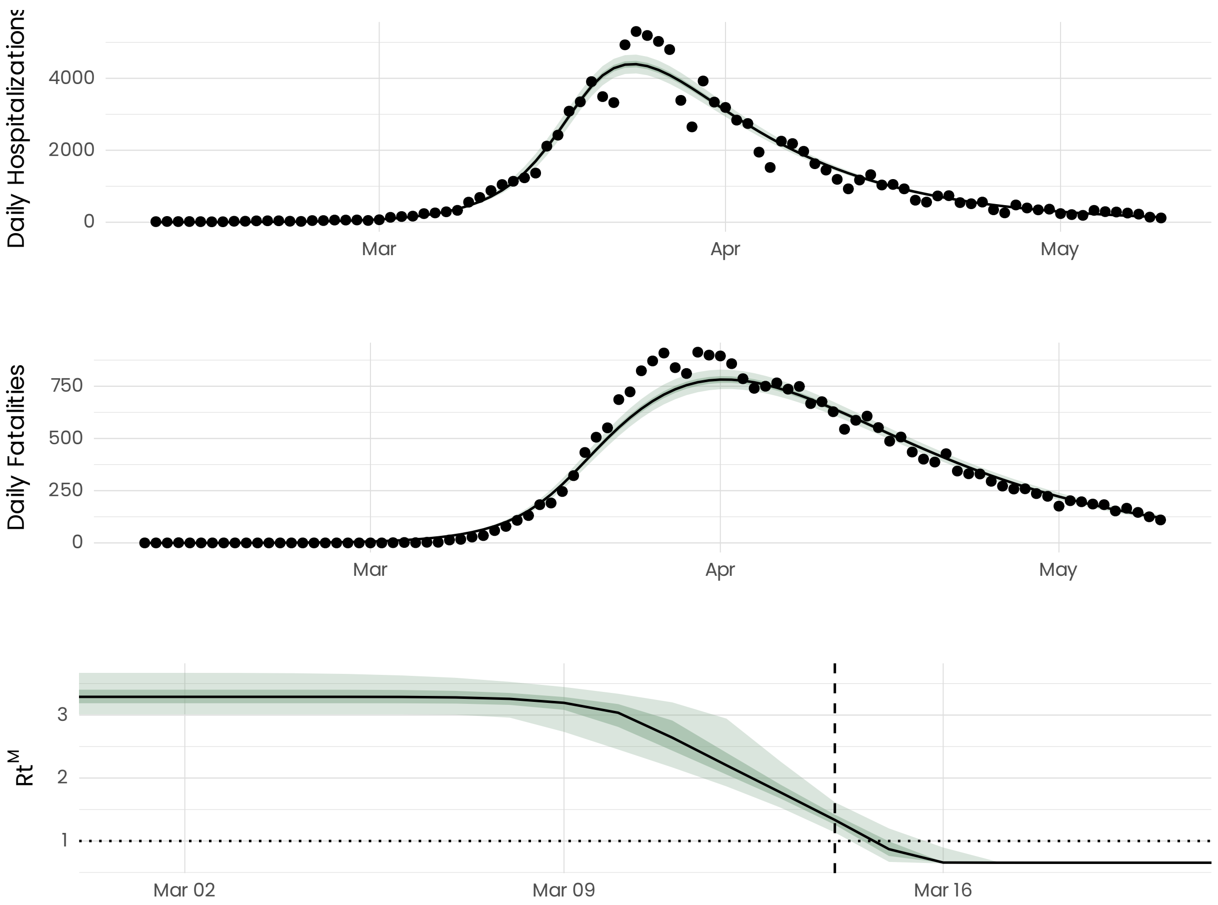

Figure S16: Top and middle panel show the adjustment for the daily hospitalizations and fatalities as Rt^M can also reach a stable value after lockdown. The bottom panel shows the inferred form of Rt^M. We find the inferred parameters I0, R1 and R2 as 1120 (CI: 210 – 1590), 3.29 (CI: 3.01 – 3.67) and 0.66 (CI: 0.64 – 0.67), respectively. The decrease is initiated on March 10 (CI: 6 – 12). Rt^M reaches a stable value between 0 and 2 days after lockdown was implemented. The median is found half a day after the implementation of lockdown. The relatively small credible interval supports our assumption that Rt^M reached a stable value on the day of lockdown. Furthermore, the rest of the inferred parameter are very similar to the ones presented in the main text.

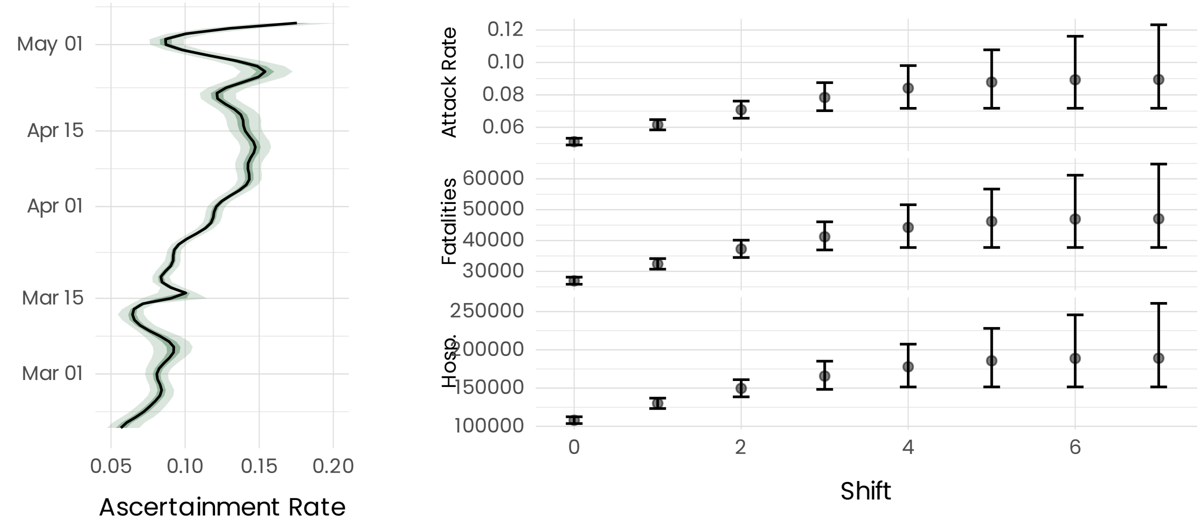

Figure S17: The left panel shows the evolution of the ascertainment rate working as Rt^M can also reach a stable value after lockdown. The right panel indicates the results of the counterfactual scenarios. In this case, the initiation of the epidemic response is shifted towards the day Rt^M reaches a stable value, which is not necessarily on the day of lockdown. For an epidemic response that was only initiated on the day Rt^M reaches the value R2 we find an attack rate of 8.9 % (CI: 7.12 – 13.3), a total number of fatalities of 47'000 (CI: 37'800 – 69'900) and hospitalizations of 189'000 (CI: 151'500 – 280'600). Also here the results are very similar to the ones presented in the main text. The main difference is that the credible intervals allow for a higher attack rate and thus total number of fatalities and hospitalizations.

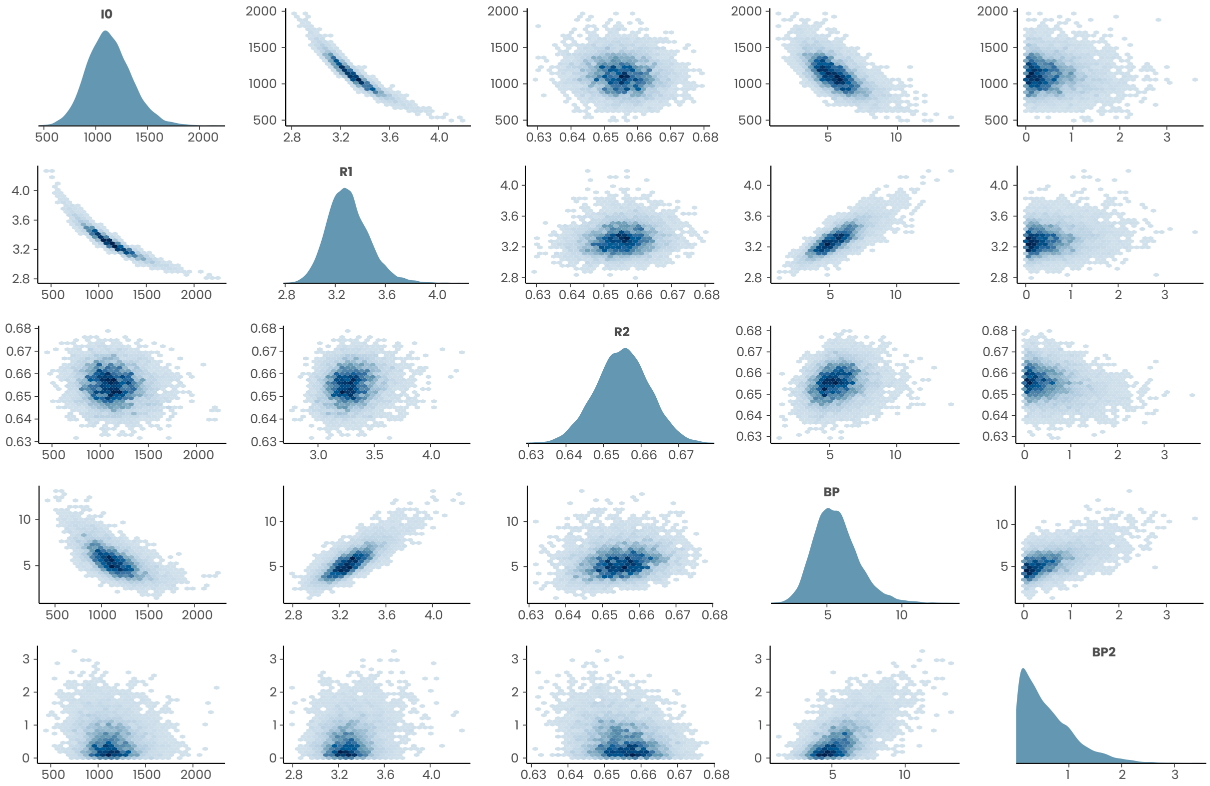

Figure S18: Posterior distributions and correlations as Rt^M can also reach a stable value after lockdown (fAfter). Looking at the posterior of fAfter we see that posterior peaks closely to lockdown, supporting our assumption in the main text.

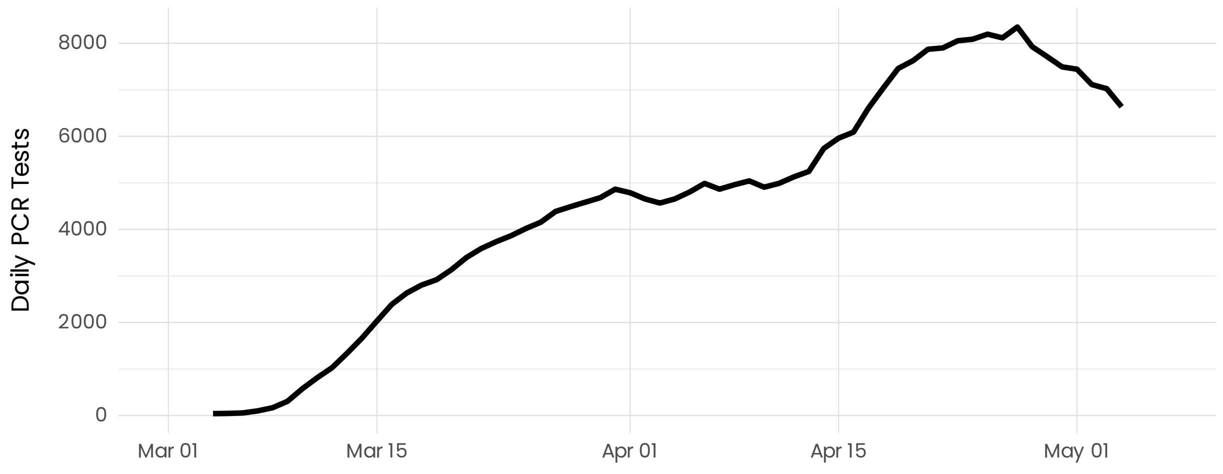

Figure S19: Number of daily PCR tests performed in Catalonia [**?**]. We observe a strong increase in the middle of April. Very likely this increase in test capacity contributed to a temporary increase in the reproduction number in the beginning of April. This is also visible in Fig.2A through a temporary increase in reported cases in the middle of April.

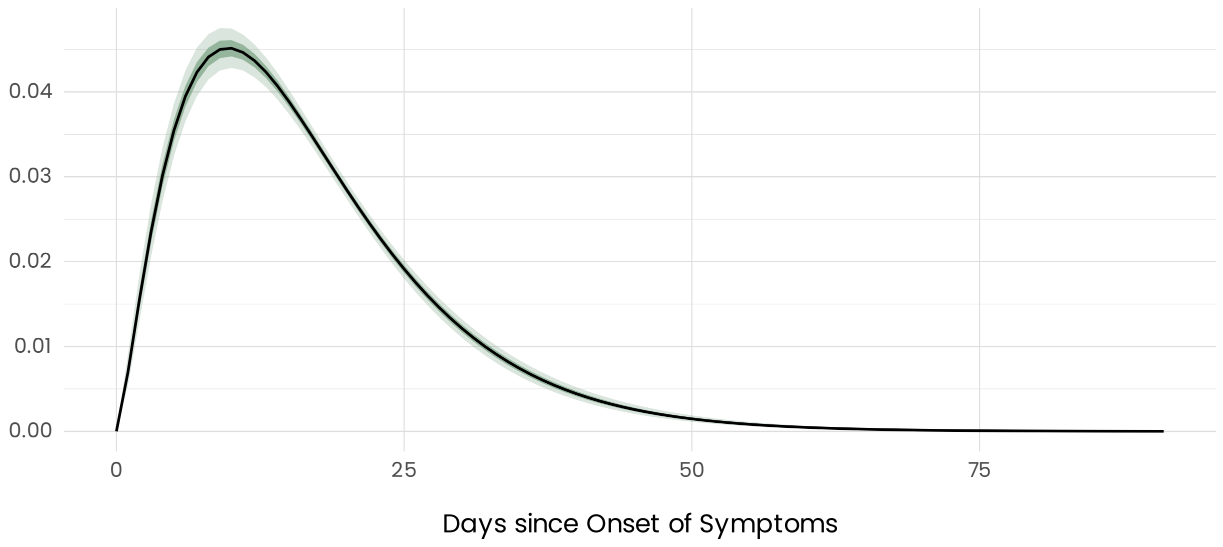

Figure S20: Posterior for the distribution between onset of symptoms and death. The median of the distribution is found as 14.5 days (CI: 13.7 – 15.4). The shape and scale factor are given by 2.36 (CI: 2.18 – 2.42) and 7.1 (CI: 6.5 – 7.9).

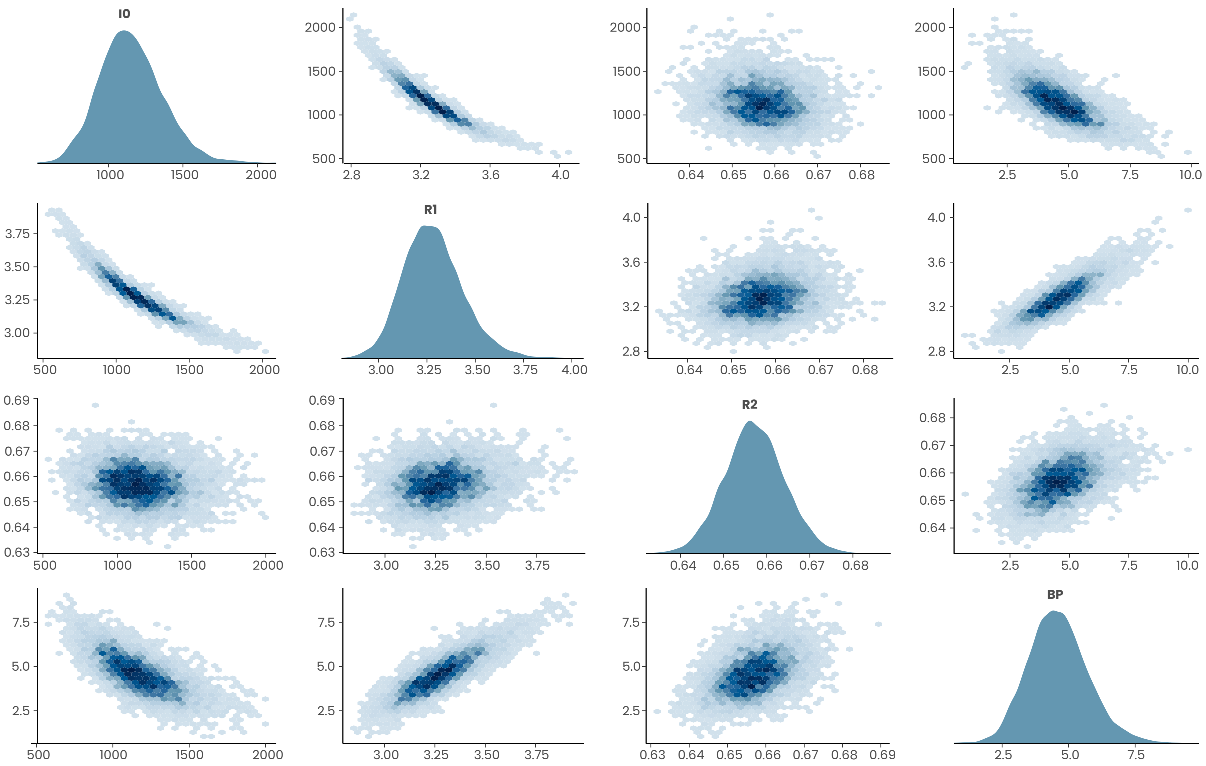

Figure S21: Posterior distributions and correlations for the parameters I0, R1, R2 and BP that defines how many days before lockdown the epidemic response was initiated.

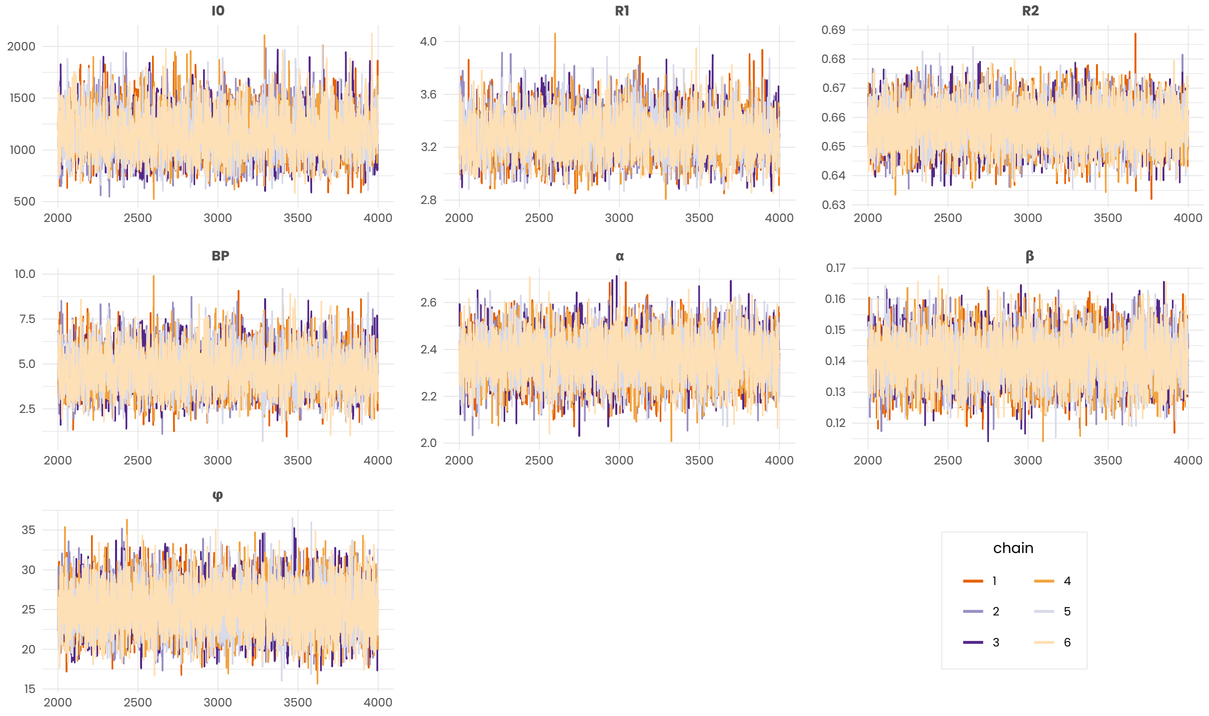

Figure S22: Trace plots for the 6 different chains for all parameters that were fitted. The parameters α and β express the shape and the inverse of the scale factor for the gamma distribution that describes time between symptom onset and death. The parameter ϕ, refers to the dispersion of the negative binomial likelihood for hospitalizations and fatalities.

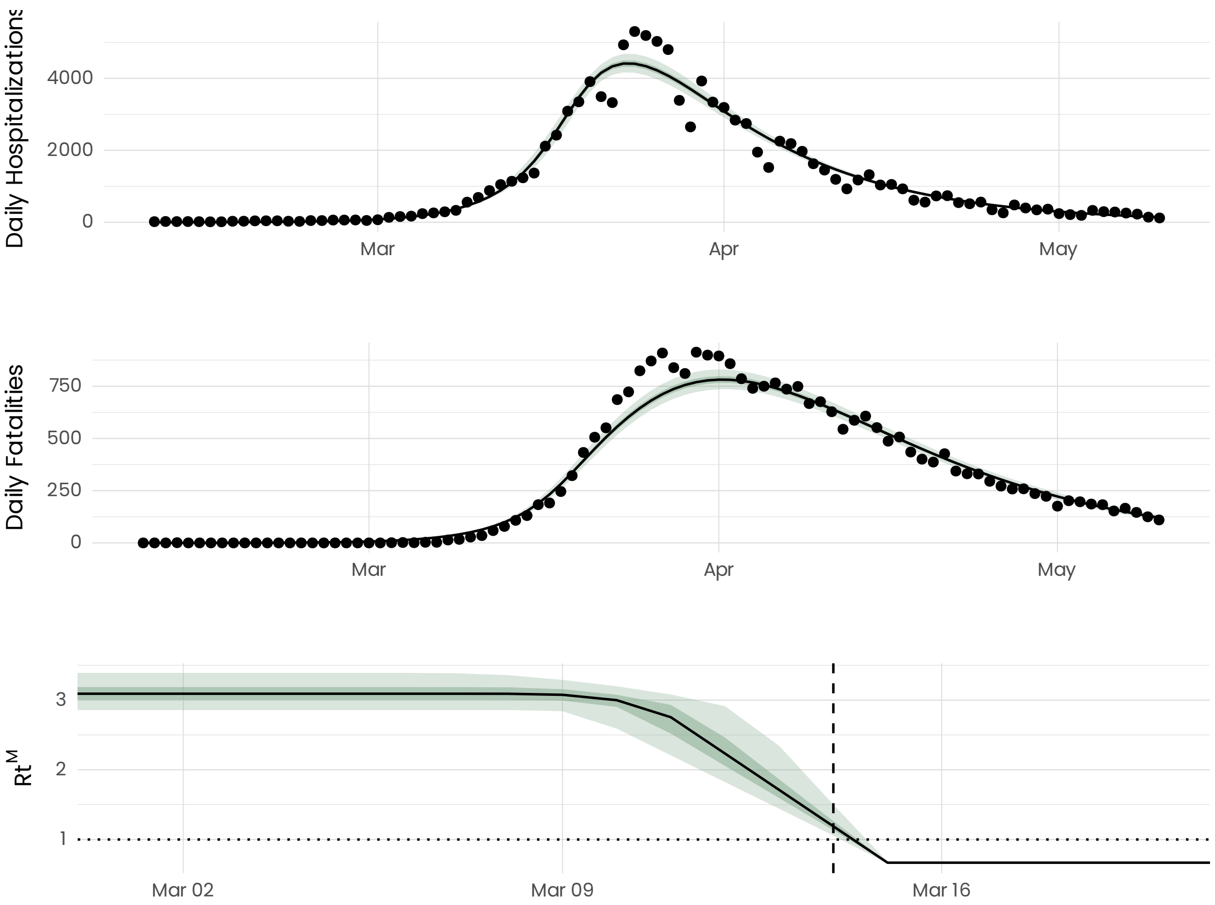

Figure S23: Top and middle panel show the adjustment for the daily hospitalizations and fatalities with a generation time distribution taken from Ferretti et al. [**?**]. The generation time corresponds to a Weibull distribution with a mean of 5.0 days and standard deviation of 1.9 days. The bottom panel shows the inferred form of Rt^M. We find the inferred parameters I0, R1 and R2 as 1060 (CI: 720 – 1500), 3.09 (CI: 2.86 – 3.39) and 0.67 (CI: 0.65 – 0.67). The decrease is initiated on March 10 (CI: 8 – 12). In this sense, the results are very similar to the ones obtained with the generation interval from Ganyani et al.[**?**].

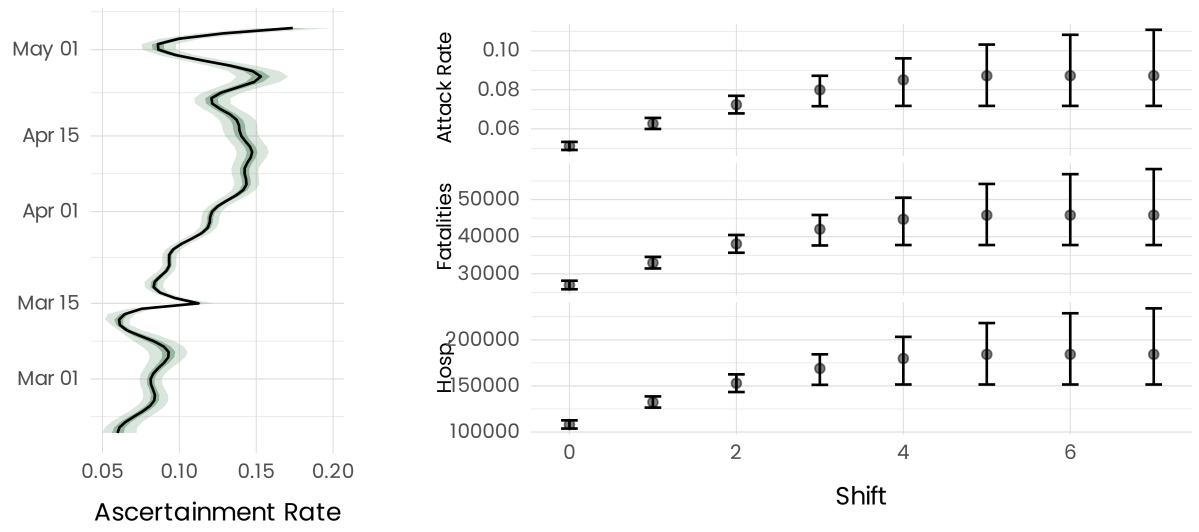

Figure S24: The left panel shows the evolution of the ascertainment rate working with the generation time of Ferretti et al. [**?**]. The right panel indicates the results of the counterfactual scenarios. For an epidemic response that was only initiated on the day of the lockdown we find an attack rate of 8.7 % (CI: 7.1 – 11.1), a total number of fatalities of 45’800 (CI: 37’800 – 58’300) and hospitalizations of 184’300 (CI: 151’500 – 234’700). Also here the results are very similar to the ones obtained with the generation time from Ganyani et al. [**?**].
